# Development of SSR markers related to salinity resistance based on transcriptomic sequences in *Medicago sativa*

**DOI:** 10.1371/journal.pone.0336528

**Published:** 2025-11-14

**Authors:** Rugang Yu, Xin Chen, Hui Zhang, Qiting Zhang, Xinyi Chen, Yanqiu Dong, Liwei Chen, Daniel Basigalup, Guoliang Wang, Xueling Du

**Affiliations:** 1 College of Life Sciences, Huaibei Normal University, Huaibei, Anhui, China; 2 Anhuii Province Key Laboratory of Pollutant Sensitive Materials and Environmental Remediation, Huaibei, Anhui, China; 3 Institute of Leisure Agriculture, Shandong Academy of Agricultural Sciences, Jinan, Shandong, China; KGUT: Graduate University of Advanced Technology, IRAN, ISLAMIC REPUBLIC OF

## Abstract

Alfalfa (*Medicago sativa*) is an important perennial forage crop that exhibits wide cultivar variations in salinity tolerance. Simple sequence repeats (SSRs) in a transcriptome can realize targeted markers that are directly related to target traits. However, SSR markers related to specific traits, especially salinity tolerance traits in alfalfa, are rarely reported worldwide. This study aimed to investigate the distribution characteristics of SSR loci and explore the key SSR loci related to salinity-tolerant genes in alfalfa. For this purpose, we conducted transcriptomic analysis of roots and leaves from GIB (G, high salinity-tolerant) and LS (L, high salinity-sensitive) plants under 0 and 200 mM NaCl treatments, which yielded 129,563 unigenes. A total of 38,370 SSR loci were identified and distributed in 28,039 unigenes, and the frequency of SSR occurrence in each locus was 4.43 kb. Among all the SSR motifs, mononucleotide (67.32%), trinucleotide (15.61%), and dinucleotide (14.53%) were the major repeated types, and the forms of A/T, AG/CT, AAG/CTT, AC/GT, AT/AT and AAC/GTT were the most frequent motifs. Meanwhile, 23,159 primer pairs of SSRs were designed for marker development in alfalfa. Among the 28,039 SSR-containing unigenes, 1,947 unigenes were found to be salinity-responsive differentially expressed unigenes (DEUs) and/or DEUs between the two cultivars. Interestingly, 188 DEUs were identified and found to be involved in ion transport, metabolite biosynthesis, ROS regulation, signaling pathway, and transcription regulation, which were all related to salinity tolerance. Notably, six out of 211 SSR loci identified based on 188 SSR-containing DEUs were validated as polymorphic SSR markers with clear amplified bands, which they exhibited high polymorphism (polymorphism information content: 0.640–0.807). Therefore, these SSR markers could be further used for authenticity identification and genetic analysis. The six SSRs were used to classify four alfalfa varieties with different salinity tolerance into three groups. The high salinity-sensitive variety LS was placed in group I, the high tolerant varieties GIB and GN5 formed group II, and the sensitive variety GN3 was included in group III. This grouping was consistent with prior evaluations of salinity tolerance. Therefore, the six SSRs may be associated with salinity tolerance in alfalfa. These findings not only provide an efficient tool for the large-scale development of markers related to specific traits but also lay a foundation for genetic analysis in alfalfa.

## Introduction

Alfalfa (*Medicago sativa* L.), which is a member of the Fabaceae family, is a forage crop cultivated worldwide; it is renowned as the “Queen of forages” because of its superior nutritive value and functional properties [1,2]. Soil salinity is among the critical environmental problems influencing alfalfa yield [3]. The screening and breeding of alfalfa varieties with salinity tolerance is critical for improving and utilizing salinized soil. Salinity tolerance in alfalfa has been known as a complex quantitative trait that is regulated by numerous genes. Notably, a significant variability in salinity tolerance exists among alfalfa varieties [3–6]. Therefore, the salinity tolerance among alfalfa varieties needs to be explored. However, the intricate salinity tolerance mechanism of alfalfa and its allogamous autotetraploid [7] cause difficulty in distinguishing the salinity tolerance among varieties.

Molecular markers are derived from defined sites within genes or regulatory sequences of plant genomes, which can be used to discriminate between and within species at the DNA level [8] Among various marker types, simple sequence repeats (SSRs) have become a crucial tool in genetic studies because of their high reproducibility, high polymorphism, codominance, and easy amplification through polymerase chain reaction (PCR) [9,10]. SSRs are short tandemly repeated sequences with motifs ranging from 1 to 6 base pairs (bp), which are distributed in coding and non-coding regions throughout the plant genomes [11]. In plants, SSR markers have been extensively employed in genetic diversity assessment [12,13], germplasm resource identification and classification [14], and trait association analysis [10,15]. For example, Li et al. utilized 15 SSR markers to identify the genetic diversity of 25 *Ilex asprella* resources, which could be divided into three populations [12]. Nie et al. used 30 randomly selected SSRs to perform marker–trait association analysis and identified 7 significant associations with drought tolerance-related traits in *Miscanthus* [15]. In alfalfa, the application of SSR markers focuses primarily on the analysis of genetic diversity [16] and identification of germplasm resources [17]. However, SSR markers related to specific traits, especially salinity tolerance trait in alfalfa, are rarely reported worldwide.

SSRs can be divided into two categories, namely, genomic SSRs and expressed sequence tag SSRs, which originate from genomic and transcriptomic sequences, respectively [10,18]. Compared with the traditional mining SSRs from genomic sequences, the developed SSR markers from transcriptomic sequences were considered a simple and effective method with low cost and less labor [14,19]. Currently, the discovery and mining of SSR loci through transcriptomic sequences have been successfully applied to many plant species [20,21]. For instance, in *Auricularia heimuer*, 53 polymorphic EST-SSRs were identified from transcriptome sequencing, of which 13 SSR markers were employed to analyze the genetic relationships of 52 *A. heimuer* germplasms [20]. In *Fagopyrum esculentum*, 20,756 EST-SSR loci within the transcriptomic sequences were detected, and 224 SSR primer pairs employed them to identify the genetic relationships of 48 *F. esculentum* varieties [21]. However, mining of SSR markers through transcriptomic sequences is rarely reported in alfalfa.

In this study, we developed SSR markers related to salinity tolerance by transcriptome sequencing. The primary aims of this study are to (i) explore the gene expression changes induced by NaCl in two alfalfa varieties with different salinity tolerance; (ii) identify the SSR-containing differentially expressed unigenes (DEUs) related to the salinity stress tolerance of alfalfa; (iii) develop SSR markers associated with salinity tolerance; and (iv) validate whether the developed SSR markers can be applied to identify salt tolerance traits in alfalfa. These findings will facilitate the development of SSR markers, genetic analysis, and molecular breeding studies in alfalfa.

## Materials and methods

### Plant materials and stress treatments

Our previous studies showed that Gibraltar (GIB) and Gannong No.5 (GN5) were high salinity-tolerant varieties, Gannong No.3 (GN3) was a salinity-sensitivity variety, and LS1405 (LS) was a variety of high salinity sensitivity [5,22]. Therefore, two varieties with significant differences in salt tolerance, namely, GIB and LS, were used for transcriptome sequencing to identify key genes related to salinity stress tolerance and develop SSR markers linked to this trait. In addition, two salinity-tolerant varieties (GIB, GN5) and two salinity-sensitive varieties (GN3, LS) were selected to validate the newly developed SSR markers. Seeds of GN5, GN3, LS, and GIB were procured commercially from the Institute of Leisure Agriculture (Jinan, China), Purple Posture Co., Ltd. (Bengbu, China), Beijing S&G Eco-Tech Co., Ltd. (Beijing, China), and Beijing Best Grass Industry Co., Ltd. (Beijing, China), respectively. In our experiment, pot trials were performed in a greenhouse at Huaibei Normal University (Huaibei, Anhui, China) under controlled conditions (25 ± 2°C with a 16/8 h light/dark cycle). Seeds of the varieties were surface sterilized in a 0.1% HgCl_2_ solution and then directly planted in sand-filled pots. After one week sowing, seedlings were thinned to eight uniform plants/pot irrigated with a 100 mL Hoagland nutrient solution (pH 5.8). Two weeks after sowing, the seedlings of GN5 and GN3 continued to be cultured with a nutrient solution, while the seedlings of GIB and LS were treated with a 100 mL nutrient solution supplemented with 0 and 200 mM NaCl, respectively. The nutrient solutions were replaced every two days. The NaCl concentrations were set according to the results of a preliminary experiment, which showed a significant difference in growth between GIB and LS under 200 mM NaCl treatment. Four weeks after sowing, root and leaf samples of GIB and LS were selected for transcriptome sequencing. Moreover, the leaf samples of GIB, GN5, GN3 and LS under NaCl-free treatment were selected to validate the newly developed SSR markers. All collected samples were flash-frozen in liquid nitrogen and stored at −80°C. Equal amount of root/leaf samples from four randomly selected individual plants (per pot) from each treatment were pooled and collected as a replicate. Two biological replicates were analyzed for transcriptome sequencing.

### Transcriptome sequencing and SSR loci mining

Total RNA was isolated from root and leaf tissues of GIB (G) and LS (L) alfalfa plants treated with 0 and 200 mM NaCl by using Trizol^®^ Reagent (Invitrogen, Carlsbad, CA, USA) according to the instructions of the manufacturer. The total amounts and integrity of RNA were assessed using the Bioanalyzer 2100 system (Agilent, CA, USA). A total of 16 cDNA libraries of roots (i.e., G_0_R1_, G_0_R2_, G_200_R1_, G_200_R2_, L_0_R1_, L_0_R2_, L_200_R1_, and L_200_R2_) and leaves (i.e., G_0_L1_, G_0_L2_, G_200_L1_, G_200_L2_, L_0_L1_, L_0_L2_, L_200_L1_, and L_200_L2_) were constructed and sequenced on the Illumina NovaSeq 6000 platform (Novogene, Beijing, China). After sequencing, clean reads were procured from the raw reads by removing the adapter sequences, N bases, and low-quality reads. Then, using Trinity (v2.6.6) [23] software, the clean reads were assembled to the reference unigene sequences for further analysis. Finally, unigenes were functionally annotated by BLAST searches against NR, Nt, Pfam, SwissProt, GO, COG/KOG, and KEGG databases.

Potential SSR loci were mined using MISA 1.0 software with the following parameters: motif lengths ranging from 1 to 6 bp, and minimum repeat counts of 10 (mono-nucleotide repeat motifs), 6 (dinucleotide repeat motifs), and 5 (all other motif types).

### Identification and enrichment analysis of DEUs containing SSR loci

Read counts of each unigene were quantified by RSEM [24]. Then, read counts of SSR-containing unigenes based on a negative binomial distribution were used for differential expression analysis among root groups (G_0_R_, G_200_R_, L_0_R_, and L_200_R_) and leaf groups (G_0_L_, G_200_L_, L_0_L_, and L_200_L_) using the DESeq2 R package (1.20.0) [25]. DEUs were defined as those with |log_2_ (fold-change)| > 1 and *P*_*adj*_ < 0.05. Functional enrichment of SSR-containing DEUs was analyzed using GOseq (v1.10.0) and KOBAS (v2.0.12) for GO and KEGG pathways (*P*_*adj*_ < 0.05).

### SSR primer design and PCR amplification

SSR primers were designed using Primer 3 (v2.5.5) software with the following criteria: a primer length of 18–27 bp, GC content of 40%–55%, annealing temperature (TM) ranging from 57°C to 62°C, a ΔTm (difference in annealing temperature between primers) of ≤ 5°C, and a PCR product size of 100–280 bp.

The SSR markers were validated as follows: (1) DNA was extracted from four alfalfa varieties with different salinity tolerance under NaCl-free treatment using M5 Plant Genomic DNA Kit (Mei5bio, Beijing, China), and an ultra-micro spectrophotometer (Aurora-900) was utilized for the DNA quantification; (2) 11 randomly selected SSR primer pairs (S1 Table) were used to verify the validity of the SSR primers by PCR with GIB material at DNA level; (3) SSR-containing DEUs related to salinity tolerance regulation were selected to develop markers related to salinity tolerance in alfalfa, and the developed SSR markers were validated by PCR with GIB, GN5, GN3, and LS materials at DNA level; (4) PCR amplification was conducted using 2X M5 HiPer plus Taq HiFi PCR mix (Mei5bio, Beijing, China) in a 10 μL reaction mixture, which consisted of 5 μL PCR Mix, 1 μL DNA (20 ng/μL), 0.5 μL forward primer (10 mM), 0.5 μL reverse primer (10 mM), and 3 μL ddH_2_O; (5) PCR products were separated by 8% polyacrylamide gel electrophoresis and visualized by silver staining.

### Genomic localization of SSR-containing DEUs related to salinity tolerance

The cultivated alfalfa genomic sequence was downloaded from figshare (https://figshare.com/articles/dataset/genome_fasta_sequence_and_annotation_files/12327602) [26]. First, unigenes related to salinity tolerance were aligned to the alfalfa genome using local BLASTN implemented in BioEdit. Genes were retained if the alignment showed greater than 80% and an *e*-value < 1e^−50^. The chromosomal localizations of the SSR-containing genes related to salinity tolerance were extracted from the GTF database and visualized using TBtools (v2.305) software.

### SSR data analysis

A 1–0 matrix dataset representing the presence (1) or absence (0) in the banding profile of SSR markers in four alfalfa varieties was constructed. Parameters related to genetic diversity (Na, Ne, H, and I) were computed using PopGene 32. The polymorphism information content (PIC) of each SSR marker was determined by PIC_CALC v.0.6. UPMGA cluster analysis was performed using NTSYS v.2.10e.

## Results

### RNA sequencing and assembly

The cDNA libraries constructed from the roots and leaves of two alfalfa varieties (NaCl/control RNA samples) were sequenced. Deep sequencing yielded between 21.27–22.84 million raw reads per library, of which 20.69–22.19 million were clean reads. Then, these clean reads were assembled into 129,563 unigenes with an average length of 959 bp and an N50 length of 1,303 bp. Among them, 117,159 unigenes were annotated according to the public databases. The raw transcriptome data generated in this study are openly available in Genome Sequence Archive (GSA) in the China National Center for Bioinformation (https://ngdc.cncb.ac.cn/gsa, No.: PRJCA019338).

### Comprehensive SSR identification

A total of 28,039 unigene sequences containing 38,370 potential SSRs with a distribution frequency of 21.64% were identified from 129,563 unigene sequences (Table 1). Among them, 7,288 unigene sequences contained more than one SSR, and 2,962 SSRs were present in compound formation (Table 1). In addition, six types of SSRs, including mono-, di-, tri-, tetra-, penta-, and hexanucleotide repeat motifs, were identiﬁed from the transcriptome data. The frequency of SSRs based on the number of repeat motif differed (Table 2). Among them, mononucleotide (67.32%) repeats were the most abundant, followed by trinucleotide (15.61%), dinucleotide (14.53%), tetranucleotide (1.45%), hexanucleotide (0.55%), and pentanucleotide (0.54%) repeat units (Table 2).

**Table 1:** Results of SSR search in alfalfa transcriptome.

Iterm	Numbers
Total size of examined sequences/bp	124,239,797
Total number of identified SSRs	38,370
Frequency of occurrence/%	21.64
Number of SSR containing sequences	28,039
Number of sequences containing more than 1 SSR	7288
Number of SSRs present in compound formation	2962

**Table 2:** Frequency of SSR types in the alfalfa transcriptome.

Repeat motif types	Repeat numbers							Total	%
	5	6	7	8	9	10	>10		
Mo	0	0	0	0	0	9335	16,495	25,830	67.32
Di	0	1578	861	623	439	321	1752	5574	14.53
Tri	3148	1329	610	327	243	132	202	5991	15.61
Tetra	327	145	52	8	7	3	14	556	1.45
Penta	153	38	3	6	2	2	5	209	0.54
Hexa	156	16	16	10	5	4	3	210	0.55
Total	3784	3106	1542	974	696	9797	18,471	38,370	
%	9.86	8.09	4.02	2.54	1.81	25.53	48.14		

**Fig 1:**
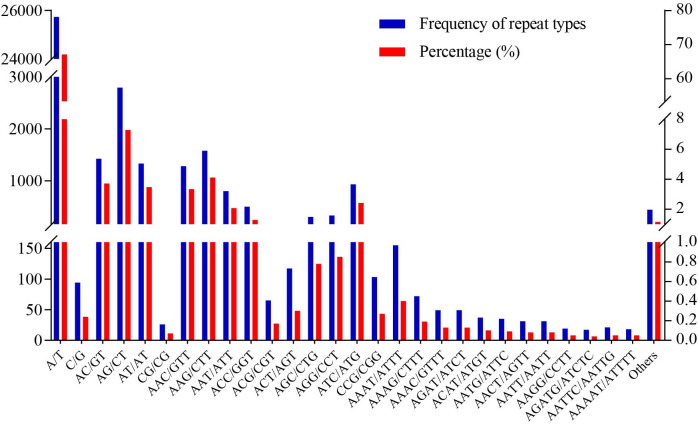
Frequency distribution of SSRs based on motif types.

A total of 196 different SSR motif types were identified from alfalfa transcriptome. Among them, A/T was the most abundant found motif, which accounted for 67.07%; AG/CT (7.28%), AAG/CTT (4.11%), AC/GT (3.71%), AT/AT (3.47%), and AAC/GTT (3.34%) were also abundant. The largest numbers of 28 SSR motif types of SSR in alfalfa transcriptome are shown in Fig 1.

### Design and validation of SSR primers

**Fig 2:**
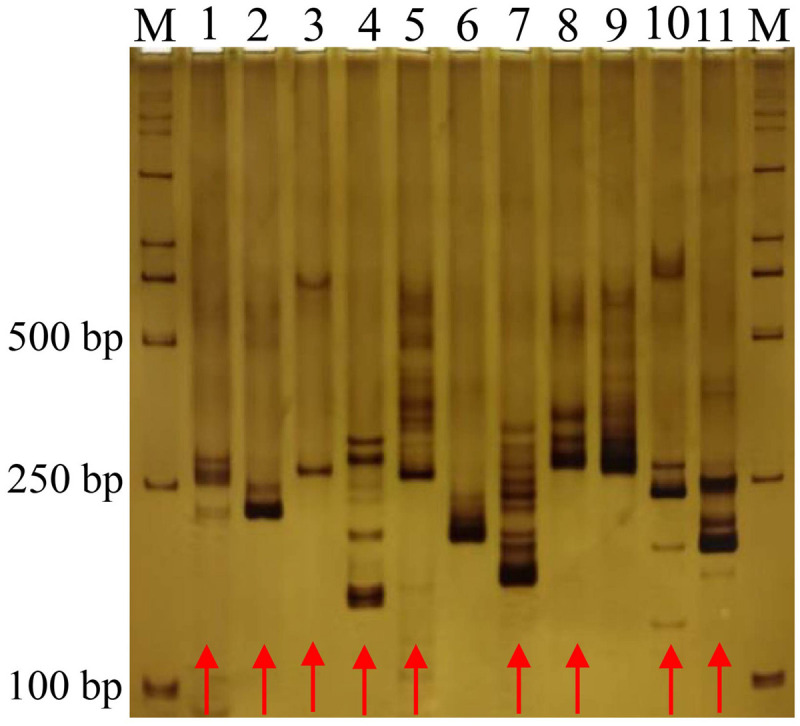
Amplification results of 11 different primers in Gibraltar. M: DL 2000 marker; the number 1–11 represent random primer 1–11; the red arrow indicates the target strip.

A total of 22,445 unigenes were successfully screened from 28,039 unigenes with SSR loci, and 23,159 primer pairs of SSRs were designed (S2 Table). Among them, 11 primer pairs (S2 Table) were randomly screened for PCR amplification. Among them, 9 SSR primer pairs successfully amplified clear bands in GIB variety, and the primer conversion rate was 81.82% (Fig 2). This result suggests that the SSR primers could be used for further analysis.

### Identification of SSR loci-containing DEUs

**Fig 3:**
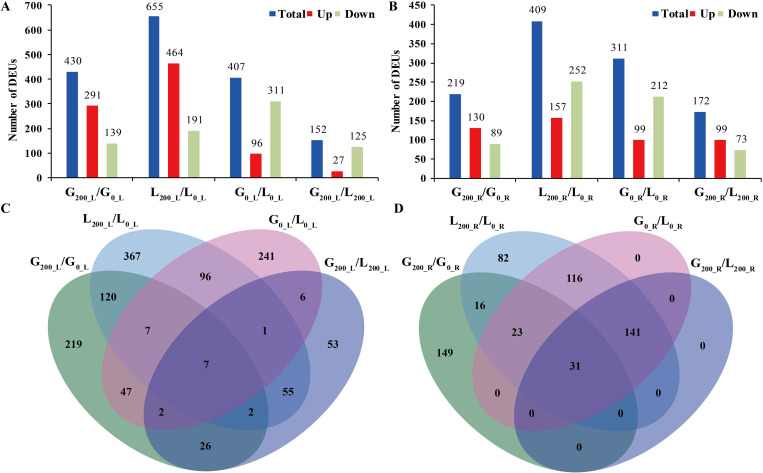
Analysis of DEUs in two alfalfa varieties under salinity treatment. **(a)** Number of DEUs in leaf. **(b)** Number of DEUs in root. **(c)** Venn diagrams of DEUs in leaf. **(d)** Venn diagrams of DEUs in root.

Pairwise comparison analysis for each unigene was conducted between GIB and LS (G_0_L_/L_0_L_, G_0_R_/L_0_R_, G_200_L_/L_200_L_, and G_200_R_/L_200_R_) or between 0 and 200 mM NaCl treatments (G_200_L_/G_0_L_, G_200_R_/G_0_R_, L_200_L_/L_0_L_, and L_200_R_/L_0_R_). The results showed that 1,947 SSR-containing unigenes were differentially regulated in eight comparisons (S3 Table). Of which, 430, 655, 407, 152, 219, 409, 311, and 172 DEUs were identified in G_200_L_/G_0_L_, L_200_L_/L_0_L_, G_0_L_/L_0_L_, G_200_L_/L_200_L_, G_200_R_/G_0_R_, L_200_R_/L_0_R_, G_0_R_/L_0_R_, and G_200_R_/L_200_R_, respectively (Figs 3A and 3B). The number of upregulated genes induced by NaCl treatment was more than that of downregulated genes in G_200_L_/G_0_L_, L_200_L_/L_0_L_, G_200_R_/G_0_R_ and G_200_R_/L_200_R_. Regardless for GIB or LS, more DEUs were identified in leaves than in the roots. Meanwhile, LS showed greater transcriptional changes than GIB either in leaves or roots. Furthermore, 136 and 70 DEUs were common NaCl-responsive genes in leaves and roots of the comparison groups, respectively. A total of 16 DEUs in leaves and 172 DEUs in roots were jointly regulated in G_0_/L_0_ and G_200_/L_200_. Moreover, 294 and 149 specific NaCl-responsive genes were detected in the GIB leaves and roots, respectively (Figs 3C and 3D).

### Functional classification of the SSR-containing DEUs by gene ontology

**Fig 4:**
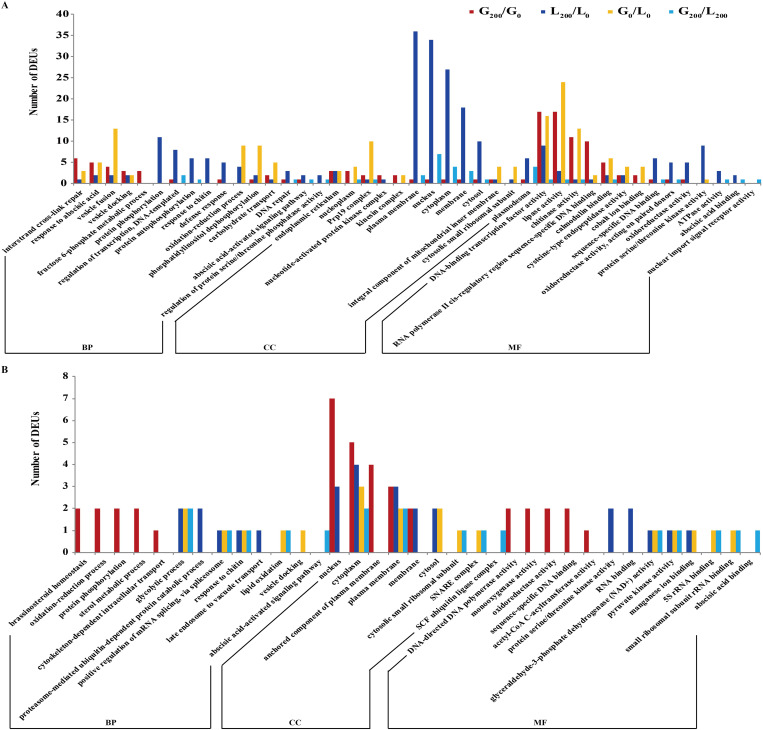
Gene Ontology classification of DEUs in leaves. **(a)** and roots **(b)**. The top 5 abundant GO terms in BP (biological process), CC (cellular component) and MF (molecular function) for G_200_/G_0_, L_200_/L_0_, G_0_/L_0_ and G_200_/ L_200._

Gene ontology (GO) annotation results of NaCl-responsive DEUs between the two varieties were significantly different either in leaves or roots. Among 1,947 DEUs, 330 (116, 196, 91, and 24 unigenes in G_200_L_/G_0_L_, L_200_L_/L_0_L_, G_0_L_/L_0_L_, and G_200_L_/L_200_L_, respectively) and 62 (43, 23, 14, and 12 unigenes in G_200_R_/G_0_R_, L_200_R_/L_0_R_, G_0_R_/L_0_R_, and G_200_R_/L_200_R_, respectively) DEUs were assigned to 441 and 173 GO terms in leaves (S4A Table) and roots (S4B Table), respectively. A total of 132, 61, 43, and 39 GO terms were obtained from G_200_R_/G_0_R_, R_200_R_/R_0_R_, G_0_R_/R_0_R_, and G_200_R_/R_200_R_, respectively (S4B Table and Fig 4B), while this value reached 248, 286, 152, and 66 GO terms in G_200_L_/G_0_L_, L_200_ L_/L_0_L_, G_0_L_/L_0_L_, and G_200_L_/L_200_L_, respectively (S4A Table and Fig 4A). The results of GO enrichment analysis (*P*-value ≤ 0.05) are listed in S5 Table. For GIB, the most enriched GO terms of NaCl-responsive DEUs included 17 (leaves) and 29 (roots) subcategories related to biological processes (e.g., fructose 6-phosphate metabolic process and brassinosteroid homeostasis), 14 (leaves) and 4 (roots) subcategories linked to cellular component (e.g., nucleotide-activated protein kinase complex, and anchored component of plasma membrane), and 17 (leaves) and 27 (roots) subcategories associated with molecular function(e.g., single-stranded telomeric DNA binding and DNA-directed DNA polymerase activity). NaCl -responsive DEUs of LS were assigned to biological processes (e.g., response to stress and glycolytic process), cellular component (e.g., plasma membrane and integral component of membrane), and molecular function (e.g., DNA-binding transcription factor activity and manganese ion binding) (S5 Table). For DEUs between GIB and LS, 8 (leaves) and 10 (roots) GO terms of biological process (e.g., potassium ion transport, defense response to fungus, glycolytic process, and abscisic acid-activated signaling pathway), 8 (leaves) and 2 (roots) GO terms of cellular component (e.g., plasmodesma, nuclear chromosome, spliceosomal complex, and SNARE complex), and 9 (leaves) and 10 (roots) GO terms of molecular function (e.g., proteasome binding, potassium ion transmembrane transporter activity, pyruvate kinase activity, and abscisic acid binding) were most enriched under NaCl stress (S5 Table).

### KEGG -based analysis of metabolic pathways associated with SSR-containing DEUs

A total of 75 (G_200_L_/G_0_L_),105 (L_200_L_/L_0_L_), 61 (G_0_L_/L_0_L_), 13 (G_200_L_/L_200_L_), 29 (G_200_R_/G_0_R_), 19 (L_200_R_/L_0_R_), 10 (G_0_R_/L_0_R_), and 7 (G_200_R_/L_200_R_) DEUs were respectively assigned to 76, 83, 50, 18, 47, 26, 17, and 12 pathways, which were classified into five categories: metabolism, genetic information processing, environmental information processing, cellular processes, and organismal systems (S6 Table). Among which, 15, 28, 16, 7, 12, 6, 4, and 6 significantly enriched pathways (*P*-value ≤ 0.05) were identified in G_200_L_/G_0_L_, L_200_L_/L_0_L_, G_0_L_/L_0_L_, G_200_L_/L_200_L_, G_200_R_/G_0_R_, L_200_R_/L_0_R_, G_0_R_/L_0_R_, and G_200_R_/L_200_R_, respectively (Table 3). For NaCl-responsive DEUs, the top two significantly enriched pathways were brassinosteroid biosynthesis and biosynthesis of secondary metabolites in G_200_R_/G_0_R_, proteasome and biosynthesis of amino acids in L_200_R_/L_0_R_, biosynthesis of secondary metabolites and flavonoid biosynthesis in G_200_L_/G_0_L_, and biosynthesis of secondary metabolites and MAPK signaling pathway in L_200_L_/L_0_L_. For DEUs between GIB and LS, mismatch repair, DNA replication, nucleotide excision repair, and homologous recombination were the pathways commonly enriched in G_0_L_/L_0_L_ and G_200_L_/L_200_L_. Glycolysis/gluconeogenesis, biosynthesis of amino acids, and linoleic acid metabolism were the pathways commonly enriched in G_0_R_/L_0_R_ and G_200_R_/L_200_R_. Furthermore, the pathways of glycolysis/gluconeogenesis, biosynthesis of amino acids, and carbon metabolism had the most significant enrichment in G_200_R_/L_200_R_. Meanwhile, cysteine and methionine metabolism, mismatch repair, DNA replication, nucleotide excision repair, and homologous recombination were the pathways enriched in G_200_L_/L_200_L_.

**Table 3:** Significantly enriched KEGG pathways for the DEUs (*P-value* ≤ 0.05).

Pathway term	DEUs tested				*P*-value				Pathway ID
	G_200_/G_0_	L_200_/L_0_	G_0_/L_0_	G_200_/L_200_	G_200_/G_0_	L_200_/L_0_	G_0_/L_0_	G_200_/L_200_	
**Significantly enriched KEGG pathways for the DEUs in leaves**									
Metabolic pathways	37	55	20	6	0.0013	0.0003	0.4355	0.6460	mtr01100
Biosynthesis of secondary metabolites	32	39	13	3	0.0000	0.0000	0.1421	0.6249	mtr01110
Carbon metabolism	3	7	1	–	0.3559	0.0495	0.8423	–	mtr01200
2-Oxocarboxylic acid metabolism	1	4	2	–	0.3551	0.0055	0.0575	–	mtr01210
Ascorbate and aldarate metabolism	2	3	3	–	0.0731	0.0324	0.0073	–	mtr00053
Nitrogen metabolism	–	3	–	–	–	0.0118	–	–	mtr00910
Glycerophospholipid metabolism	–	3	3	–	–	0.1440	0.0389	–	mtr00564
Fatty acid degradation	2	4	–	–	0.0772	0.0061	–	–	mtr00071
Glycerolipid metabolism	1	4	1	–	0.5234	0.0299	0.4758	–	mtr00561
alpha-Linolenic acid metabolism	4	4	1	–	0.0021	0.0095	0.3632	–	mtr00592
Sphingolipid metabolism	–	1	2	–	–	0.3212	0.0213	–	mtr00600
Biosynthesis of unsaturated fatty acids	2	1	–	–	0.0183	0.2665	–	–	mtr01040
Cysteine and methionine metabolism	3	7	2	2	0.0624	0.0006	0.1839	0.0304	mtr00270
Arginine and proline metabolism	1	4	3	–	0.4495	0.0152	0.0164	–	mtr00330
Phenylalanine metabolism	2	–	1	–	0.0518	–	0.2690	–	mtr00360
Arginine biosynthesis	–	3	–	–	–	0.0126	–	–	mtr00220
Alanine, aspartate and glutamate metabolism	–	4	–	–	–	0.0058	–	–	mtr00250
Valine, leucine and isoleucine biosynthesis	1	3	2	–	0.1277	0.0015	0.0069	–	mtr00290
Valine, leucine and isoleucine degradation	3	5	2	–	0.0080	0.0005	0.0480	–	mtr00280
Lysine degradation	1	3	–	–	0.3458	0.0298	–	–	mtr00310
Histidine metabolism	2	2	–	–	0.0247	0.0538	–	–	mtr00340
Cyanoamino acid metabolism	1	4	–	–	0.4766	0.0196	–	–	mtr00460
beta-Alanine metabolism	1	3	1	–	0.3315	0.0262	0.2960	–	mtr00410
Selenocompound metabolism	–	1	–	1	–	0.2074	–	0.0446	mtr00450
Taurine and hypotaurine metabolism	–	–	–	1	–	–	–	0.0362	mtr00430
Pantothenate and CoA biosynthesis	1	2	2	–	0.2606	0.0812	0.0297	–	mtr00770
Nicotinate and nicotinamide metabolism	–	2	–	–	–	0.0284	–	–	mtr00760
Thiamine metabolism	2	–	–	–	0.0148	–	–	–	mtr00730
Carotenoid biosynthesis	–	–	2	–	–	–	0.0284	–	mtr00906
Terpenoid backbone biosynthesis	3	1	–	–	0.0105	0.4910	–	–	mtr00900
Brassinosteroid biosynthesis	2	1	–	–	0.0065	0.1623	–	–	mtr00905
Limonene and pinene degradation	1	2	–	–	0.0760	0.0074	–	–	mtr00903
Phenylpropanoid biosynthesis	6	5	–	–	0.0217	0.2345	–	–	mtr00940
Flavonoid biosynthesis	5	6	–	–	0.0010	0.0012	–	–	mtr00941
Isoflavonoid biosynthesis	3	4	1	1	0.0010	0.0003	0.1503	0.0549	mtr00943
Protein processing in endoplasmic reticulum	3	4	6	–	0.4194	0.4925	0.0186	–	mtr04141
SNARE interactions in vesicular transport	–	3	3	–	–	0.0159	0.0034	–	mtr04130
Homologous recombination	2	2	5	2	0.1813	0.3336	0.0007	0.0232	mtr03440
Nucleotide excision repair	2	2	5	2	0.1585	0.2973	0.0005	0.0197	mtr03420
DNA replication	2	2	5	2	0.1266	0.2444	0.0002	0.0151	mtr03030
Mismatch repair	2	2	5	2	0.1033	0.2039	0.0001	0.0120	mtr03430
Plant hormone signal transduction	7	10	4	–	0.0300	0.0176	0.2534	–	mtr04075
MAPK signaling pathway – plant	6	11	12	2	0.0032	0.0000	0.0000	0.0667	mtr04016
Plant-pathogen interaction	4	5	7	1	0.1094	0.1500	0.0012	0.4197	mtr04626
Circadian rhythm – plant	2	4	–	–	0.0731	0.0055	–	–	mtr04712
**Significantly enriched KEGG pathways for the DEUs in roots**									
Biosynthesis of secondary metabolites	12	5	3	2	0.0028	0.1231	0.2944	0.3473	mtr01110
Carbon metabolism	1	2	2	2	0.6058	0.0969	0.0553	0.0250	mtr01200
Biosynthesis of amino acids	3	3	2	2	0.0459	0.0104	0.0415	0.0185	mtr01230
Glycolysis/ Gluconeogenesis	–	2	2	2	–	0.0280	0.0152	0.0066	mtr00010
Glycerophospholipid metabolism	–	2	1	–	–	0.0188	0.1396	–	mtr00564
alpha-Linolenic acid metabolism	2	–	–	–	0.0224	–	–	–	mtr00592
Linoleic acid metabolism	–	1	1	1	–	0.0637	0.0464	0.0302	mtr00591
Purine metabolism	2	2	2	1	0.0529	0.0185	0.0100	0.0919	mtr00230
Valine, leucine and isoleucine degradation	2	–	–	–	0.0136	–	–	–	mtr00280
Thiamine metabolism	2	–	–	–	0.0031	–	–	–	mtr00730
Terpenoid backbone biosynthesis	2	–	–	–	0.0165	–	–	–	mtr00900
Brassinosteroid biosynthesis	2	–	–	–	0.0013	–	–	–	mtr00905
Ribosome	1	3	2	2	0.7545	0.0451	0.1113	0.0525	mtr03010
SNARE interactions in vesicular transport	–	1	1	1	–	0.0785	0.0574	0.0375	mtr04130
Proteasome	1	2	–	–	0.1908	0.0066	–	–	mtr03050
Homologous recombination	2	1	1	–	0.0459	0.1733	0.1285	–	mtr03440
Nucleotide excision repair	2	1	1	–	0.0392	0.1600	0.1183	–	mtr03420
DNA replication	2	1	1	–	0.0304	0.1403	0.1035	–	mtr03030
Mismatch repair	2	1	1	–	0.0242	0.1249	0.0919	–	mtr03430

### SSR-containing DEUs related to salinity tolerance in alfalfa

**Fig 5:**
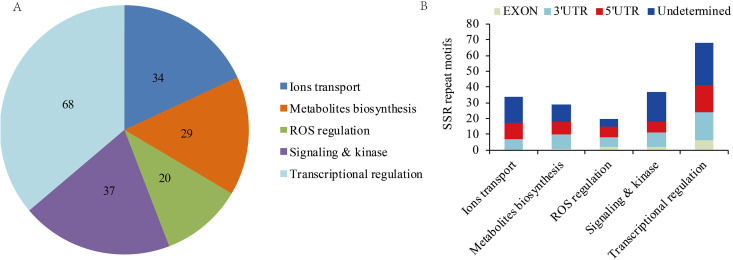
Functional classification of SSR containing DEUs related to salinity stress tolerance (a) and location of SSRs in different regions of the salinity stress tolerance genes in each functional class (b).

Signal transductions, ion transport, metabolite biosynthesis, ROS regulation, and transcriptional regulation are important processes for plants to combat salinity stress [27,28]. Based on GO and KEGG pathway analyses, 34 DEUs encoding ion transport-related proteins, 29 DEUs related to metabolite biosynthesis, 20 DEUs associated with ROS regulation, 37 DEUs linked to signaling and kinase, and 68 DEUs mapped to different transcription factor families were identified in this study (S7A Table and Fig 5A). These DEUs were divided into three distinct expression patterns: (I) 139 salinity-responsive DEUs, (II) 36 salinity-responsive DEUs that were differentially expressed between GIB and LS, and (III) 13 DEUs differentially expressed between GIB and LS (S7B Table).

The distribution of SSR motifs within the gene regions (exons, 3’UTR, and 5’UTR) of the five functional classifications was analyzed (Fig 5B). The locations of the largest number of SSR motifs in three regions were transcriptional regulation genes (6 exons, 18 3’UTR, and 17 5’UTR), followed by signaling and kinase (2) and ROS regulation (2) genes located in the exonic region, signaling and kinase (9) and metabolite biosynthesis (9) genes in the 5’UTR region, and ion transport (10) genes in the 5’UTR region (Fig 5B).

### Development of SSR markers linked to salinity tolerance genes in alfalfa

**Fig 6:**
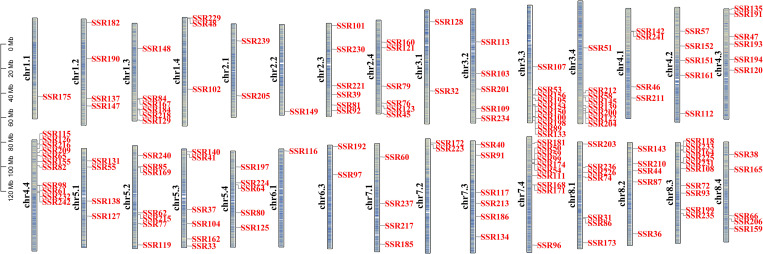
Genomic localization of SSR markers on 30 *M. sativa* chromosomes.

**Fig 7:**
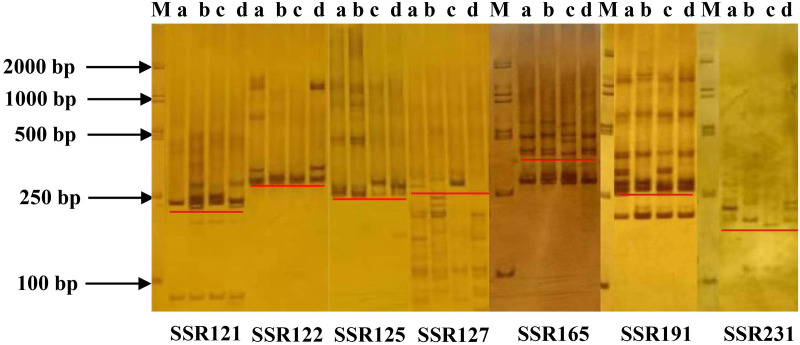
Amplification of seven SSR primer pairs using genomic DNA of four alfalfa varieties as template. The four bars **(a–d)** as a group, and the sequence of each group is GN3, GIB, LS and GN5.

Combined with the SSR loci data from transcriptome sequencing, the SSR loci information of the 188 previously identified salinity tolerance-related unigenes were screened out and extracted. Among them, some unigenes contained two or more SSR loci; a total of 211 SSR primer pairs designed based on standard PCR primer criteria were synthesized for validation across four alfalfa varieties exhibiting contrasting salinity tolerance: GN3, GN5, GIB, and LS (S8 Table). A total of 200 out of 211 SSR loci were successfully localized on the 30 alfalfa chromosomes (Fig 6). Among 211 primer pairs, 110 primer pairs were detected with amplification products, of which 7 primer pairs (SSR121, SSR122, SSR125, SSR127, SSR165, SSR191, and SSR231) had relatively clear amplified bands. Their amplified bands differed in GN3, GN5, GIB, and LS (Fig 7), which could be used to distinguish different varieties. By searching the alfalfa genome annotation file (GTF/GFF) (S8 Table), we found that SSR122 and SSR125 loci were located in the 3’UTR region of the protein phosphatase 2C 12 (*PP2C12*, chr3.4) and *PP2C25* (chr5.4) gene, respectively; SSR165 was located in the 5’UTR region of the ethylene-responsive transcription factor (*ERF026*, chr8.4) gene; SSR121, SSR127, SSR191, and SSR231 were located in the undetermined regions of the leucine-rich repeat receptor-like serine/threonine-protein kinase (*LRR-RLK*, chr2.4), *PP2C2* (chr5.1), heat shock 70 kDa protein (*HSP70*, chr4.3), and two-component response regulator (*ARR5*, chr8.3) gene, respectively (Table 4).

**Table 4:** Development the salinity stress tolerance related seven SSR markers.

Primer ID	Gene ID	Annotation	Gene name	(Motif) repeats	Amplified product (bp)	Position	Chr.
SSR121	Cluster-27126.42501	Leucine-rich repeat receptor-like serine/threonine-protein kinase	*LRR-RLK/At3g14840*	(T)14	230–270	undetermined	chr2.4
SSR122	Cluster-27126.45536	Protein phosphatase 2C 12,	*PP2C12*	(A)10	270–280	3’UTR	chr3.4
SSR125	Cluster-27126.43113	Protein phosphatase 2C 25	*PP2C25*	(T)11	249–260	3’UTR	chr5.4
SSR127	Cluster-27126.43458	Protein phosphatase 2C 2	*PP2C2*	(A)11,(A)15, (TTG)6	250–260	undetermined	chr5.1
SSR165	Cluster-27126.29621	Ethylene-responsive transcription factor ERF026	*ERF026*	(CTT)5	280–400	5’UTR	chr8.4
SSR191	Cluster-27126.57746	Heat shock 70 kDa protein	*HSP70*	(AG)10	241–300	undetermined	Chr4.3
SSR231	Cluster-27126.24781	Two-component response regulator ARR5	*ARR5*	(AAT)9	189–230	undetermined	Chr8.3

### Genetic diversity and cluster analysis of four alfalfa varieties with contrasting salinity tolerance based on six polymorphic SSR markers

Among the seven SSR markers previously tested, except for SSR127, the six other polymorphic markers were used to assess the genetic diversity of GN3, GN5, GIB, and LS. A total of 31 bands were generated by the amplification, and 50 alleles were detected from six SSR markers (mean = 1.6667 alleles/marker) (S9 Table). Among them, SSR125 and SSR165 amplified the largest observed number of alleles (Na). The number of effective alleles (Ne) ranged from 1.0000 to 2.0000, with an average value of 1.3780. The average value of Nei’s gene diversity (H, range of 0.0000–0.5000) and Shannon’s information index (I, range of 0.0000–0.6931) were 0.2326 and 0.3526, respectively (S9 Table). The number of PIC ranged from 0.640 to 0.807 (mean = 0.7225) (S9 Table).

**Fig 8:**
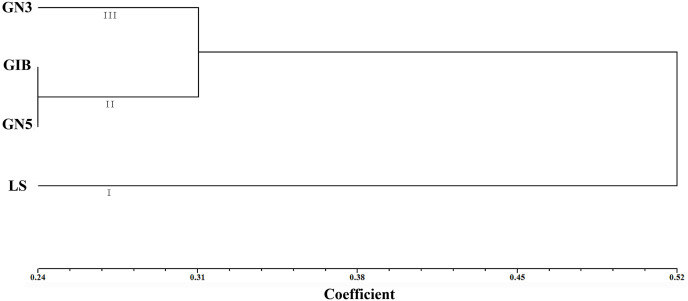
Cluster analyses of four alfalfa varieties differing in salinity tolerance.

Based on the genetic distance coefficient, Unweighted Pair Group Method with Arithmetic Mean (UPGMA) cluster analysis of six polymorphic SSR markers data showed that the four different alfalfa varieties could be clustered into three main groups at a genetic distance coefficient of 0.31. The first group comprised LS (high salinity sensitivity), the second group included GIB and GN5 (high salinity tolerance), and the third group consisted of GN3 (salinity sensitivity) (Fig 8).

## Discussion

Salinity stress is a key environmental factor limiting the growth of plants worldwide. Alfalfa, as a vital forage crop, is relatively susceptible to salinity stress [29]. Notably, a great variability in salinity tolerance exists among alfalfa varieties [22,30,31]. However, accurate identification and differentiation between varieties is limited due to the self-incompatibility and cross-pollination behavior of autotetraploid species [32]. SSR loci are distributed throughout plant genomes and have been effectively used to analyze genetic diversity [16] and identify genotypes [17,33] in tetraploid alfalfa. Although many SSR markers have been developed in alfalfa, the development of salinity tolerance-associated SSR markers in alfalfa remains rarely reported.

### Transcriptome sequencing as an effective method for developing SSR markers in alfalfa

Transcriptome sequencing has been effectively and rapidly utilized to identify key stress-tolerant genes [10,34], and develop SSR markers in many plant species, such as *Anoectochilus roxburghii* [35], *Miscanthus* [15], and *Chimonanthus praecox* [36]. Nie et al. detected 16,566 gene-associated SSR loci from the transcriptomic sequences of *Miscanthus* under drought stress, which promoted the development of SSR markers related to drought resistance [15]. Liu et al. identified 64,440 SSRs through transcriptome sequencing of *C. praecox*, and 75 polymorphic SSR markers were used to analyze the genetic relationships of 12 *C. praecox* varieties [36]. In this study, 129,563 unigenes were obtained through transcriptome sequencing on the roots and leaves of GIB and LS treated with salinity stress (Table 1). Using the MISA script, 38,370 SSR loci distributed among 28,039 unigenes were detected, with a distribution frequency of 21.64% (Table 1). This frequency was lower than that reported for *Polygonatum odoratum* (29.47%) [14] but higher than that of *Anoectochilus emeiensis* (11.18%) [37]. Among the SSR repeat units, mononucleotide (67.32%) repeats were the most abundant, followed by trinucleotide (15.61%), and dinucleotide (14.53%) repeat units (Table 2). This finding was similar to the sequencing results of *P. odoratum* [14] and *Chrysanthemum* [10] but different from those of *Miscanthus* [15] and *C. praecox* [36]. In our study, A/T (67.07%) was the most abundant mononucleotide repeat (Fig 1), which was similar to *Chrysanthemum* [10]. Meanwhile, we found that AG/CT (7.28%) repeats were the dominant dinucleotide motifs (Fig 1), which was also consistent with those reported for other species such as *Miscanthus* [15] and *C. praecox* [36]. In trinucleotide repeats, AAG/CTT (4.11%) was the more frequent (Fig 1), which was consistent with those reported for some dicotyledonous plants such as *C. praecox* [36] and *Carex rigescens* [38]. By contrast, this finding was different from those reported for some monocotyledons plants such as *Miscanthus* [15] and *Dendrocalamus brandisii* [39]. These differences may be due to methodological variations, differences in database availability, and species-specific characteristics.

A total of 23,159 alternate primer pairs of SSRs were designed based on the transcriptome data in this study (S1 Table). The amplification efficiency of randomly selected primer pairs was 81.8%, which was higher than that in *P. odoratum* (60.2%) [14] and *Miscanthus* (75.3%) [15]. The abovementioned results indicated that the SSR primers developed based on transcriptome sequencing in this study are effective and feasible.

### Development and application of SSR markers associated with salinity stress tolerance in alfalfa

Functional markers are derived from polymorphic sites within genes or regulatory sequences that are directly linked to phenotypic trait variation [8]. SSRs identified in transcriptome data are located in coding and non-coding regions and often correlate with specific functions; thus, mining SSR markers from transcriptome data realize targeted identification of trait-associated loci [10,40]. For example, Shi et al. developed eight polymorphic SSR markers related to flower colors based on transcriptomic sequences of genes involved in carotenoid or anthocyanin synthesis [10]. To identify SSR markers associated with salinity stress tolerance in alfalfa, 1,947 SSR-containing DEUs were identified in pairwise comparisons (S3 Table). GO and KEGG pathway annotation showed that the biosynthesis of secondary metabolites was most enriched in G_200_L_/G_0_L_ and L_200_L_/L_0_L_ (Table 3). Among the 1,947 DEUs, 188 SSR-containing DEUs were identified to be related to ion transport (34), metabolite biosynthesis (29), ROS regulation (20), signalling pathway (37), and transcription regulation (68) (S7A Table and Fig 5A). These processes are known to regulate plant salinity tolerance [27,28], which indicates that they may contribute to the differences in salinity tolerance between the two alfalfa varieties.

To validate whether the abovementioned results can be used as SSR markers to identify alfalfa varieties with different salinity tolerance, the SSR loci information of the 188 previously identified salinity-responsive related DEUs were screened out and extracted, and 211 SSR primer pairs (S8 Table) were synthesized for validation in GN5, GIB, GN3, and LS [5,22]. A total of seven markers produced clear amplification bands, with six (SSR121, SSR122, SSR125, SSR165, SSR191 and SSR231) showing polymorphism (Fig 7). Therefore, they could be used to distinguish different varieties. For instance, SSR231, SSR121, and SSR122, SSR121 and SSR125, SSR122 and SSR165, and SSR122 and SSR191 can distinguish four different alfalfa varieties with 100% accuracy. SSR127 and SSR165 can distinguish LS from the other alfalfa varieties (Fig 7). Interestingly, these SSRs originated from the transcript sequences of *LRR-RLK*, *PP2C12*, *PP2C25*, *ERF026*, *HSP70*, and *ARR5* genes (Table 4). *LRR-RLKs*, *PP2Cs*, *ERFs*, and *HSPs* are important regulators of plant responses to abiotic stress. Transcriptome sequencing results showed that *PP2C12*, *PP2C25*, *ERF026* in roots, and *ARR5* in leaves were upregulated by NaCl treatment in GIB, while in LS, they were unchanged. Meanwhile, *LRR-RLK* was downregulated by NaCl treatment in LS, while in GIB, it was unchanged, *and HSP70* was upregulated by NaCl treatment in both varieties (S3 Table). Overexpression of *OsSTLK* (a member of LRR-RLK) [41], *PnLRR-RLK27* [42], *PagERF021* [43], and *MdHSP70–38* [44] enhanced the salinity stress tolerance of transgenic plants through regulating ROS scavenging system. Overexpression of *AtARR22* [45] and *AtARR5* [46] genes in *Arabidopsis* enhanced the freezing tolerance of transgenic lines by improving cell membrane integrity. *PP2CA* together with *ABI1* inhibited SnRK2.4 activity and regulated plant responses to salinity [47], and overexpression of *BpPP2C1* in *Betula platyphylla* improved the salinity tolerance of transgenic lines, and knockout of *BpPP2C1* exhibited sensitive to salt stress [48]. Therefore, we speculate that these genes may serve as marker genes for distinguishing the four alfalfa varieties with contrasting salinity tolerance.

PIC values represent the informativeness of molecular marker [10]. Here, the PIC values of six SSRs ranged from 0.640 to 0.807, and the average PIC value was 0.7225 (PIC > 0.5) (S9 Table), which suggests that the newly developed SSR markers in this study could be further used for variety authentication and genetic analysis in alfalfa.

Meanwhile, polymorphic SSR markers have been widely used for classification in many plants, such as *Chrysanthemum* [10], chrysanthemum [13], and *Arctium lappa* [49]. In this study, through UPGMA clustering based on genetic distances, the six newly developed SSR markers classified four alfalfa varieties into three groups: group I (LS), group II (GIB and GN5), and group III (GN3) (Fig 8). This finding is similar to our previous results on salinity tolerance in alfalfa, which categorized the varieties into three groups: high salinity tolerance (GIB and GN5), salinity sensitivity (GN3), and high salinity sensitivity (LS) [5,22]. The combination of salt tolerance, PCR, and cluster analyses indicated a potential association between the six newly developed SSR markers and salinity tolerance in alfalfa.

## Conclusions

In this study, 38,370 SSR loci were detected from transcriptome sequencing, and 23,159 primer pairs of SSRs were successfully designed based on these loci. Furthermore, 188 SSR-containing DEUs were involved in ion transport, metabolite biosynthesis, ROS regulation, signaling pathway, and transcription regulation, which were all related to salinity tolerance. Six polymorphic SSR markers derived from transcriptome data were validated and found to be involved in signaling pathway and ROS regulation. Cluster analysis grouped the four alfalfa varieties into three groups: high salinity-sensitive variety LS (group I), high salinity-tolerant varieties GIB and GN5 (group II), and salinity-sensitive variety GN3 (group III). This classification pattern suggests a potential association between the six SSR markers and salinity tolerance in alfalfa. Whether these markers are functionally linked to salinity-tolerant genes and whether they can reliably identify salinity tolerance in alfalfa needs further verification. This study provides a solid foundation for the large-scale development of markers related to specific traits, genetic diversity studies, classification, and molecular-assisted breeding in alfalfa.

## Supporting information

S1 Table List of 11 SSR random primer pairs used for PCR amplification verification in GIB. (XLSX)

S2 Table List of 23,159 SSR primer pairs were designed in this study. (XLSX)

S3 Table List of DEUs in eight groups based on pairwise comparisons. (XLSX)

S4A Table The GO function classification of all DEUs in four comparisons of leaves. (XLSX)

S4B Table The GO function classification of all DEUs in four comparisons of roots. (XLSX)

S5A Table The enriched GO terms of all DEUs in four comparisons of leaves. (XLSX)

S5B Table The enriched GO terms of all DEUs in four comparisons of roots. (XLSX)

S6A Table Overview of all 104 KEGG pathways for DEUs in four comparisons of leaves. (XLSX)

S6B Table Overview of all 104 KEGG pathways for DEUs in four comparisons of roots. (XLSX)

S7A Table Listed functional classification of SSR-containing DEUs related to salinity stress tolerance. (XLSX)

S7B Table Listed classification of SSR-containing DEUs related to salinity stress tolerance based on their expression patterns. (XLSX)

S8 Table List of 211 SSR primer pairs used for the development of salinity-tolerant related SSR markers. (XLSX)

S9 Table Genetic diversity analysis with polymorphism SSR markers developed in this study. (XLSX)

## References

[1] Tlahig S, Elfalleh W. Alfalfa as a nutritional and functional food resource: applications and health benefits. Food Bioscience. 2025;68:106762. doi: 10.1016/j.fbio.2025.106762

[2] Zhang Y, Wang L. Advances in basic biology of alfalfa (*Medicago sativa* L.): a comprehensive overview. Hortic Res. 2025;12(7):uhaf081. doi: 10.1093/hr/uhaf081 40343348 PMC12058308

[3] Benabderrahim MA, Guiza M, Haddad M. Genetic diversity of salt tolerance in tetraploid alfalfa (*Medicago sativa* L.). Acta Physiol Plant. 2020;42(1). doi: 10.1007/s11738-019-2993-8

[4] Cornacchione MV, Suarez DL. Evaluation of Alfalfa (*Medicago sativa* L.) populations’ response to salinity stress. Crop Science. 2017;57(1):137–50. doi: 10.2135/cropsci2016.05.0371

[5] Yu R, Wang X, Wang G, Xu Z, Gao Q, Du X, et al. Analysis of salinity-tolerance and screening of salinity-tolerance evaluation indicators in *Medicago sativa* L. varieties at seedling stage. Acta Agrestia Sinica. 2022;30(7):1781–9. doi: 10.11733/j.issn.1007-0435.2022.07.020

[6] Huang K, Dai X, Xu Y, Dang S, Shi T, Sun J, et al. Relation between level of autumn dormancy and salt tolerance in lucerne (*Medicago sativa*). Crop & Pasture Science. 2018;69(2):194–204. doi: 10.1071/cp17121

[7] Alzahrani OR, Alshehri MA, Alasmari A, Ibrahim SD, Oyouni AA, Siddiqui ZH. Evaluation of genetic diversity among Saudi Arabian and Egyptian cultivars of alfalfa (*Medicago sativa* L.) using ISSR and SCoT markers. J Taibah Univ Sci. 2023;17(1). doi: 10.1080/16583655.2023.2194187

[8] Lübberstedt T, Zein I, Andersen JR, Wenzel G, Krützfeldt B, Eder J, et al. Development and application of functional markers in maize. Euphytica. 2005;146(1–2):101–8. doi: 10.1007/s10681-005-0892-0

[9] Powell W, Machray GC, Provan J. Polymorphism revealed by simple sequence repeats. Trends in Plant Science. 1996;1(7):215–22. doi: 10.1016/1360-1385(96)86898-1

[10] Shi Z, Zhao W, Li Z, Kang D, Ai P, Ding H, et al. Development and validation of SSR markers related to flower color based on full-length transcriptome sequencing in *Chrysanthemum*. Sci Rep. 2022;12(1):22310. doi: 10.1038/s41598-022-26664-3 36566291 PMC9789954

[11] Li T, Cai S, Cai Z, Fu Y, Liu W, Zhu X, et al. TriticeaeSSRdb: a comprehensive database of simple sequence repeats in *Triticeae*. Front Plant Sci. 2024;15:1412953. doi: 10.3389/fpls.2024.1412953 38841284 PMC11150838

[12] Li J, Quan C, Wei R, Wei F, Ma Q, Huang Y, et al. Genome-wide identification and development of SSR molecular markers for genetic diversity studies in *Ilex asprella*. Front Plant Sci. 2025;16:1582154. doi: 10.3389/fpls.2025.1582154 40487224 PMC12143170

[13] Olejnik A, Parkitna K, Kozak B, Florczak S, Matkowski J, Nowosad K. Assessment of the genetic diversity of *Chrysanthemum* Cultivars using SSR markers. Agronomy. 2021;11(11):2318. doi: 10.3390/agronomy11112318

[14] Pan G, Xie J, Qin Y, Zhang S. Development of SSR markers for genetic diversity analysis and species identification in *Polygonatum odoratum* (Mill.) Druce based on transcriptome sequences. PLoS One. 2024;19(9):e0308316. doi: 10.1371/journal.pone.0308316 39312515 PMC11419394

[15] Nie G, Tang L, Zhang Y, Huang L, Ma X, Cao X, et al. Development of SSR markers based on transcriptome sequencing and association analysis with drought tolerance in perennial grass *Miscanthus* from China. Front Plant Sci. 2017;8:801. doi: 10.3389/fpls.2017.00801 28559912 PMC5432562

[16] Yazıcılar B, Jannati G, Bezirganoglu I. Genetic variations in Turkey cultivar and ecotype *Medicago sativa* species: cytological, total protein profile, and molecular characterization. J Genet Eng Biotechnol. 2021;19(1):59. doi: 10.1186/s43141-021-00159-6 33928463 PMC8085131

[17] Azzam CR, Abd El-Naby ZM, Abd El-Rahman SS, Omar SA, Ali EF, Majrashi A, et al. Association of saponin concentration, molecular markers, and biochemical factors with enhancing resistance to alfalfa seedling damping-off. Saudi J Biol Sci. 2022;29(4):2148–62. doi: 10.1016/j.sjbs.2021.11.046 35531163 PMC9072927

[18] Wu H-B, Gong H, Liu P, He X-L, Luo S-B, Zheng X-M, et al. Large-scale development of EST-SSR markers in sponge gourd via transcriptome sequencing. Mol Breeding. 2014;34(4):1903–15. doi: 10.1007/s11032-014-0148-6

[19] Squirrell J, Hollingsworth PM, Woodhead M, Russell J, Lowe AJ, Gibby M, et al. How much effort is required to isolate nuclear microsatellites from plants?. Mol Ecol. 2003;12(6):1339–48. doi: 10.1046/j.1365-294x.2003.01825.x 12755865

[20] Jiao L, Han C, Zhu J, Zhang P, Ma Y, Dai X, et al. Transcriptome analysis and development of EST-SSR markers in the mushroom *Auricularia heimuer*. Sci Rep. 2024;14(1):12340. doi: 10.1038/s41598-024-63080-1 38811679 PMC11136984

[21] Liu Y, Fang X, Tang T, Wang Y, Wu Y, Luo J, et al. Inflorescence transcriptome sequencing and development of new EST-SSR markers in common buckwheat (*Fagopyrum esculentum*). Plants (Basel). 2022;11(6):742. doi: 10.3390/plants11060742 35336623 PMC8950064

[22] Yu R, Wang G, Yu X, Li L, Li C, Song Y, et al. Assessing alfalfa (*Medicago sativa* L.) tolerance to salinity at seedling stage and screening of the salinity tolerance traits. Plant Biol (Stuttg). 2021;23(4):664–74. doi: 10.1111/plb.13271 33884732

[23] Grabherr MG, Haas BJ, Yassour M, Levin JZ, Thompson DA, Amit I, et al. Full-length transcriptome assembly from RNA-Seq data without a reference genome. Nat Biotechnol. 2011;29(7):644–52. doi: 10.1038/nbt.1883 21572440 PMC3571712

[24] Li B, Dewey CN. RSEM: accurate transcript quantification from RNA-Seq data with or without a reference genome. BMC Bioinform. 2011;12:323. doi: 10.1186/1471-2105-12-323 21816040 PMC3163565

[25] Love MI, Huber W, Anders S. Moderated estimation of fold change and dispersion for RNA-seq data with DESeq2. Genome Biol. 2014;15(12):550. doi: 10.1186/s13059-014-0550-8 25516281 PMC4302049

[26] Chen H, Zeng Y, Yang Y, Huang L, Tang B, Zhang H, et al. Allele-aware chromosome-level genome assembly and efficient transgene-free genome editing for the autotetraploid cultivated alfalfa. Nat Commun. 2020;11(1):2494. doi: 10.1038/s41467-020-16338-x 32427850 PMC7237683

[27] Zhao S, Zhang Q, Liu M, Zhou H, Ma C, Wang P. Regulation of plant responses to salt stress. Int J Mol Sci. 2021;22(9):4609. doi: 10.3390/ijms22094609 33924753 PMC8125386

[28] Waseem M, Muhammad Aslam M, Kumar Sahu S. Understanding the mechanistic basis of plant adaptation to salinity and drought. Funct Plant Biol. 2024;51:FP23216. doi: 10.1071/FP23216 38347662

[29] Peel MD, Anower MR, Wu Y. Breeding efficiency for salt tolerance in alfalfa. Life (Basel). 2023;13(11):2188. doi: 10.3390/life13112188 38004328 PMC10672560

[30] Fan S, Chen J, Mu J, Zhang M. Genetic diversity and salt tolerance assessment of 51 alfalfa (*Medicago sativa*) varieties under saline soil conditions. Front Sustain Food Syst. 2023;7. doi: 10.3389/fsufs.2023.1278913

[31] Feng Y, Chen Z, Chen L, Han M, Liu J, Liu Y, et al. Comprehensive evaluation of physio-morphological traits of alfalfa (*Medicago sativa* L.) varieties under salt stress. Physiol Plant. 2025;177(1):e70044. doi: 10.1111/ppl.70044 39780763

[32] Zhao Z, Zhang W, Liu Y, Li S, Yao W, Sun X, et al. De novo hydroponics system efficiency for the cuttings of alfalfa (*Medicago sativa* L.). Physiol Mol Biol Plants. 2021;27(6):1413–21. doi: 10.1007/s12298-021-00995-3 34220046 PMC8212189

[33] Annicchiarico P, Nazzicari N, Ananta A, Carelli M, Wei Y, Brummer EC. Assessment of cultivar distinctness in alfalfa: a comparison of genotyping-by-sequencing, simple-sequence repeat marker, and morphophysiological observations. Plant Genome. 2016;9(2):10.3835/plantgenome2015.10.0105. doi: 10.3835/plantgenome2015.10.0105 27898838

[34] Jin Y, Yan H, Zhu X, Yang Y, Jia J, Sun M, et al. Single-cell transcriptomes reveal spatiotemporal heat stress response in pearl millet leaves. New Phytol. 2025;247(2):637–50. doi: 10.1111/nph.70232 40415399

[35] Zhang W, Chen K, Mei Y, Wang J. De novo transcriptome assembly of *Anoectochilus roxburghii* for morphological diversity assessment and potential marker development. Plants (Basel). 2024;13(23):3262. doi: 10.3390/plants13233262 39683058 PMC11644659

[36] Liu B, Wu H-F, Cao Y-Z, Yang X-M, Sui S-Z. Establishment of novel simple sequence repeat (SSR) markers from *Chimonanthus praecox* transcriptome data and their application in the identification of varieties. Plants (Basel). 2024;13(15):2131. doi: 10.3390/plants13152131 39124249 PMC11313930

[37] Lu S. Transcriptome analysis and development of EST-SSR markers in *Anoectochilus emeiensis* (Orchidaceae). PLoS One. 2022;17(12):e0278551. doi: 10.1371/journal.pone.0278551 36472967 PMC9725121

[38] Li M, Long R, Feng Z, Liu F, Sun Y, Zhang K, et al. Transcriptome analysis of salt-responsive genes and SSR marker exploration in *Carex rigescens* using RNA-seq. J Integr Agric. 2018;17(1):184–96. doi: 10.1016/s2095-3119(17)61749-0

[39] Geng R, Xu J, Jiang J, Cheng Z, Sun M, Xia N, et al. Identification of new cultivar and different provenances of *Dendrocalamus brandisii* (Poaceae: Bambusoideae) using simple sequence repeats developed from the whole genome. Plants (Basel). 2024;13(20):2910. doi: 10.3390/plants13202910 39458856 PMC11511551

[40] Singh AK, Chaurasia S, Kumar S, Singh R, Kumari J, Yadav MC, et al. Identification, analysis and development of salt responsive candidate gene based SSR markers in wheat. BMC Plant Biol. 2018;18(1):249. doi: 10.1186/s12870-018-1476-1 30342465 PMC6195990

[41] Lin F, Li S, Wang K, Tian H, Gao J, Zhao Q, et al. A leucine-rich repeat receptor-like kinase, OsSTLK, modulates salt tolerance in rice. Plant Sci. 2020;296:110465. doi: 10.1016/j.plantsci.2020.110465 32540023

[42] Wang J, Liu S, Li C, Wang T, Zhang P, Chen K. PnLRR-RLK27, a novel leucine-rich repeats receptor-like protein kinase from the Antarctic moss *Pohlia nutans*, positively regulates salinity and oxidation-stress tolerance. PLoS One. 2017;12(2):e0172869. doi: 10.1371/journal.pone.0172869 28241081 PMC5328275

[43] Fan G, Gao Y, Wu X, Yu Y, Yao W, Jiang J, Liu H, Jiang T. Functional analysis of *PagERF021* gene in salt stress tolerance in *Populus alba* × *P. glandulosa*. Plant Genome. 2024; 17(4): e20521. doi: 10.1002/tpg2.20521 39414577 PMC11628909

[44] Han X, Song C, Fang S, Wei Y, Tian J, Zheng X, et al. Systematic identification and analysis of the *HSP70* genes reveals *MdHSP70-38* enhanced salt tolerance in transgenic tobacco and apple. Int J Biol Macromol. 2025;289:138943. doi: 10.1016/j.ijbiomac.2024.138943 39701234

[45] Kang NY, Cho C, Kim J. Inducible expression of *Arabidopsis response regulator 22 (ARR22)*, a Type-C *ARR*, in transgenic *Arabidopsis* enhances drought and freezing tolerance. PLoS One. 2013;8(11):e79248. doi: 10.1371/journal.pone.0079248 24244460 PMC3828410

[46] Shi Y, Tian S, Hou L, Huang X, Zhang X, Guo H, et al. Ethylene signaling negatively regulates freezing tolerance by repressing expression of *CBF* and type-A *ARR* genes in *Arabidopsis*. Plant Cell. 2012;24(6):2578–95. doi: 10.1105/tpc.112.098640 22706288 PMC3406918

[47] Krzywińska E, Kulik A, Bucholc M, Fernandez MA, Rodriguez PL, Dobrowolska G. Protein phosphatase type 2C PP2CA together with ABI1 inhibits SnRK2.4 activity and regulates plant responses to salinity. Plant Signal Behav. 2016;11(12):e1253647. doi: 10.1080/15592324.2016.1253647 27901636 PMC5225939

[48] Xing B, Gu C, Zhang T, Zhang Q, Yu Q, Jiang J, et al. Functional study of *BpPP2C1* revealed its role in salt stress in *Betula platyphylla*. Front Plant Sci. 2021;11:617635. doi: 10.3389/fpls.2020.617635 33519877 PMC7841333

[49] Su Y, Fu J, Xie H, Huang Z, Li Y, Luo Y, et al. SSR markers development and their application in genetic diversity of burdock (*Arctium lappa* L.) germplasm. BMC Plant Biol. 2025;25(1):196. doi: 10.1186/s12870-025-06203-8 39953403 PMC11827309

