# Supplementary material for: Development of SSR markers related to salinity resistance based on transcriptomic sequences in Medicago sativa
Source: PLoS One. 2025 Nov 14;20(11):e0336528. doi: 10.1371/journal.pone.0336528 (PMC12617910; doi:10.1371/journal.pone.0336528)
Supplement: Attachment — Submitted filename: Reviewer attachment.docx [file pone.0336528.s014.docx]

This manuscript identified SSR loci that might be related to the salinity tolerance in alfalfa through transcriptome SSR markers. This finding not only provide an efficient tool for the large-scale development of markers related to specific traits, but also lays a foundation for genetic analysis in alfalfa. While the findings from this manuscript are interesting and worth publishing, there are some points that need to be addressed. The manuscript should be accepted after the authors addressed the following questions.

1. The selection of varieties should be clear and the reasons should be explained.

2. Four varieties were selected for SSR markers, and the basis for selecting two varieties for transcriptomics sequencing was what?

3. The experimental methods are not clear. The basis for setting the salt treatment concentration needs to be elaborated in detail. The usage methods of salt treatment and nutrient solution need to be described in detail.

4. The number of varieties in the cluster analysis is too small, and the salt tolerance evaluation is not representative.
