# Supplementary material for: Development of SSR markers related to salinity resistance based on transcriptomic sequences in Medicago sativa
Source: PLoS One. 2025 Nov 14;20(11):e0336528. doi: 10.1371/journal.pone.0336528 (PMC12617910; doi:10.1371/journal.pone.0336528)
Supplement: Attachment — Submitted filename: Response to Editor and Reviewers-2025.0908.docx [file pone.0336528.s017.docx]

Dear Editor,

Thank you for giving us a chance to revise our manuscript, “***Development of SSR markers related to salinity resistance based on transcriptome sequences in Medicago sativa***” (Ms. No. **PONE-D-25-38113**), and we also thank the reviewers for their constructive suggestions. Based on these suggestions, we have made careful modifications on the text. All changes made to the text are indicated in red color in the revised manuscript. We also responded point by point to each reviewer comments as listed below.

Hope these will make it more acceptable for publication.

Should there be any questions or suggestions, please feel free to contact me by e-mail at wangguoliang@126.com.

Guoliang Wang, Ph.D. Professor

-------------------------------------------------------------------------------------------------------

Response to Editor and Reviewer Comments

**Response to Editor Comments**

**When submitting your revision, we need you to address these additional requirements.**

**1. *Please ensure that your manuscript meets PLOS ONE's style requirements, including those for file naming. The PLOS ONE style templates can be found at https://journals.plos.org/plosone/s/file?id=wjVg/PLOSOne_formatting_sample_main_body.pdf and*** [***https://journals.plos.org/plosone/s/file?id=ba62/PLOSOne_***](https://journals.plos.org/plosone/s/file?id=ba62/PLOSOne_)***formatt-***

***ting_sample_title_authors_affiliations.pdf.***

**Response:** Many thanks for the editor’s comments. We have carefully checked the full text, which meets PLOS ONE's style requirements.

**2. *PLOS ONE now requires that authors provide the original uncropped and unadjusted images underlying all blot or gel results reported in a submission’s figures or Supporting Information files. This policy and the journal’s other requirements for blot/gel reporting and figure preparation are described in detail at https://journals.plos.org/plosone/s/figures#loc-blot-and-gel-reporting-requirements and https://journals.plos.org/plosone/s/figures#loc-preparing-figures-from-image-files. When you submit your revised manuscript, please ensure that your figures adhere fully to these guidelines and provide the original underlying images for all blot or gel data reported in your submission. See the following link for instructions on providing the original image data:*** [***https://journals.plos.org/plosone/s/figures#loc-original-images-for-blots-and-gels***](https://journals.plos.org/plosone/s/figures#loc-original-images-for-blots-and-gels)***.*** ***In your cover letter, please note whether your blot/gel image data are in Supporting Information or posted at a public data repository, provide the repository URL if relevant, and provide specific details as to which raw blot/gel images, if any, are not available. Email us at plosone@plos.org if you have any questions.***

**Response:** We would like to thank the reviewer for pointing out this issue. According to your suggestion, the original uncropped and unadjusted image of SSR121, SSR122, SSR125, SSR127, SSR165, SSR191, and SSR231 has been provided as follows:

**Fig 7. Amplification of seven SSR primer pairs using genomic DNA of four alfalfa varieties as template. The four bars (a–d) as a group, and the sequence of each group is GN3, GIB, LS and GN5.**

**(1) The original uncropped and unadjusted images of SSR121, SSR122, SSR125, SSR127**

**SSR121**

**SSR122**

**SSR125**

**SSR127**

**(2) The original uncropped and unadjusted image of SSR165**

**SSR165**

**(3) The original uncropped and unadjusted image of SSR191**

**SSR191**

**(3) The original uncropped and unadjusted image of SSR231**

**SSR231**

***3. If the reviewer comments include a recommendation to cite specific previously published works, please review and evaluate these publications to determine whether they are relevant and should be cited. There is no requirement to cite these works unless the editor has indicated otherwise.***

**Response:** No.

**Response to Reviewer Comments**

**Reviewer 1:**

***This manuscript identified SSR loci that might be related to the salinity tolerance in alfalfa through transcriptome SSR markers. This finding not only provide an efficient tool for the large-scale development of markers related to specific traits, but also lays a foundation for genetic analysis in alfalfa. While the findings from this manuscript are interesting and worth publishing, there are some points that need to be addressed. The manuscript should be accepted after the authors addressed the following questions.***

**Response:** Many thanks for the reviewer’s comments. We have carefully revised the relevant contents according to your suggestion, as follows:

***1. The selection of varieties should be clear and the reasons should be explained.***
**Response:** Thank you very much for reviewer’s constructive suggestions. Our previous studies showed that Gibraltar (GIB) and Gannong No.5 (GN5) were high salinity-tolerant varieties, Gannong No.3 (GN3) was a salinity-sensitivity variety, and LS1405 (LS) was a variety of high salinity sensitivity (**Yu et al. 2021**; **Yu et al. 2022**). Therefore, two varieties with significant differences in salt tolerance, namely, GIB and LS, were used for transcriptome sequencing to identify key genes related to salinity stress tolerance and develop SSR markers linked to this trait.

Transcriptome sequencing has been effectively and rapidly utilized for the development of SSR marker in plants. SSRs can realize targeted markers directly related to target traits. Therefore, we developed SSR markers related to salinity tolerance based on the transcriptome sequencing data. Moreover, to verify whether the developed SSR markers can be applied to the identification of salt tolerance traits of alfalfa, two salinity-tolerant varieties (GIB, GN5) and two salinity-sensitive varieties (GN3, LS) were selected to validate the newly developed SSR markers.

**References**

Yu R, Wang G, Yu X, Li L, Li C, Song Y, Xu Z, Zhang J, Guan C. **Assessing alfalfa (*Medicago sativa* L.) tolerance to salinity at seedling stage and screening of the salinity tolerance traits**. Plant Biol. 2021; 23(4): 664–674.

Yu R, Wang X, Wang G, Xu Z, Gao Q, Du X, zhang Y. **Analysis of salinity-tolerance and screening of salinity-tolerance evaluation indicators in *Medicago sativa* L. varieties at seedling stage**. Acta Agrestia Sinica 2022; 30(7): 1781–1789.

Furthermore, according to your suggestions, we have added the reasons for variety selection in the manuscript. The corresponding sentences of the ***Methods*** (**Now in** **lines 128-135, 150-153**) section have been rewritten in the revised manuscript.

***2. Four varieties were selected for SSR markers, and the basis for selecting two varieties for transcriptomics sequencing was what?***

**Response:** We would like to thank the reviewer for pointing out this issue. Alfalfa is an important perennial forage crop. Salinity is among the most harmful agents that negatively influence alfalfa yield. However, the mechanism of salinity tolerance in alfalfa has not been well understood. Our previous study on salinity tolerance analysis of alfalfa showed that the GIB and GN5 were high salinity tolerance varieties, GN3 was salinity sensitivity variety, and LS was high salinity sensitivity variety (**Yu et al. 2021**; **Yu et al. 2022**). However, the mechanism of salinity tolerance in alfalfa plants remains unclear. Therefore, to identify key genes related to salinity stress tolerance, the GIB (high salinity tolerant) and LS (high salinity sensitive) varieties were used for transcriptome sequencing.

Furthermore, salinity tolerance in alfalfa has been known as a complex quantitative trait which is regulated by numerous genes. Notably, there is a significant variability in salinity tolerance among alfalfa varieties (**Benabderrahim et al. 2021**; **Yu et al. 2021**; **Yu et al. 2022**). Therefore, salinity tolerance is one of the most important breeding objectives of alfalfa. SSRs are one of the most crucial markers in genetic research and plant breeding, which can realize targeted markers directly related to target traits (**Shi et al. 2022**). However, few SSR markers related to salinity tolerance traits have been reported in alfalfa. Transcriptome sequencing has been effectively and rapidly utilized for the development of SSR marker in many plant species (**Zhang et al. 2024**; **Liu et al. 2024**). Therefore, we developed SSR markers related to salinity tolerance through the transcriptome sequencing in this study. Moreover, to verify whether the developed SSR markers can be applied to the identification of salt tolerance traits of alfalfa, we selected four varieties (GN3, GN5, GIB, LS) differing in salinity tolerant to validate SSR markers related to salinity based on transcripts participated in regulation to plant salinity tolerance.

**References**

Yu R, Wang G, Yu X, Li L, Li C, Song Y, Xu Z, Zhang J, Guan C. **Assessing alfalfa (*Medicago sativa* L.) tolerance to salinity at seedling stage and screening of the salinity tolerance traits**. Plant Biol. 2021; 23(4): 664–674.

Yu R, Wang X, Wang G, Xu Z, Gao Q, Du X, zhang Y. **Analysis of salinity-tolerance and screening of salinity-tolerance evaluation indicators in *Medicago sativa* L. varieties at seedling stage**. Acta Agrestia Sinica 2022; 30(7): 1781–1789.

Benabderrahim MA, Guiza M, Haddad M. **Genetic diversity of salt tolerance in tetraploid alfalfa (*Medicago sativa* L.)**. Acta Physiol Plant. 2020; 42: 1–11.

Shi Z, Zhao W, Li Z, Kang D, Ai P, Ding H, Wang Z. **Development and validation of SSR markers related to flower color based on full-length transcriptome sequencing in Chrysanthemum**. Sci Rep. 2022; 12(1): 22310.

Zhang W, Chen K, Mei Y, Wang J. **De novo transcriptome assembly of anoectochilus roxburghii for morphological diversity assessment and potential marker development**. Plants. 2024; 13(23): 3262.

Liu B, Wu HF, Cao YZ, Yang XM, Sui SZ. **Establishment of novel simple sequence repeat (SSR) markers from *Chimonanthus praecox* transcriptome data and their application in the identification of varieties**. Plants. 2024; 13(15): 2131.

In addition, to more accurately and precisely delineate the results of our study, the corresponding sentences of ***Introduction*** (**Now in lines 117-124**) **and *Methods*** (**Now in lines 128-135, 150-153**) sections have been rewritten in the revised manuscript.

***3. The experimental methods are not clear. The basis for setting the salt treatment concentration needs to be elaborated in detail. The usage methods of salt treatment and nutrient solution need to be described in detail.***

**Response:** Many thanks for the reviewer’s constructive suggestions. We had carefully corrected the related information according to the reviewer’s suggestions. The main corresponding revisions were listed as follows.

**In plant materials and stress treatments,** “After one week, seedlings were thinned to eight uniform plants/pot…” **was revised as** “After one week sowing, seedlings were thinned to eight uniform plants/pot irrigated with a 100 mL Hoagland nutrient solution (pH 5.8). Two weeks after sowing, the seedlings of GN5 and GN3 continued to be cultured with a nutrient solution, while the seedlings of GIB and LS were treated with a 100 mL nutrient solution supplemented with 0 and 200 mM NaCl, respectively. The nutrient solutions were replaced every two days. The NaCl concentrations were set according to the results of a preliminary experiment, which showed a significant difference in growth between GIB and LS under 200 mM NaCl treatment.” in the revised manuscript (**Now in Lines 142-150**).

***4. The number of varieties in the cluster analysis is too small, and the salt tolerance evaluation is not representative.***

**Response:** We would like to thank the reviewer for pointing out this issue. There is no rigid requirement for the minimum number of samples in cluster analysis, but the size of sample will directly affect the accuracy of cluster analysis. Just as the reviewer’s comments, if the number of samples is too small, it may also lead to inaccurate results of cluster analysis. However, cross-validation can make full use of the data and improve the accuracy of analysis with a small sample size.

In this study, based on the salt tolerance analysis of more than 30 alfalfa varieties in our previously study, we selected two varieties with high salt tolerance (GIB and GN5), one with salt sensitivity (GN3) and one with high salt sensitivity (LS), to validate whether the newly developed SSR markers can divide four varieties, and whether it is related to the salt tolerance of alfalfa. PCR analysis was carried out to detect the amplification products of 7 newly developed SSR markers (SSR121, SSR122, SSR125, SSR127, SSR165, SSR191 and SSR231). The amplified bands each of those are different in GN3, GN5, GIB and LS (Fig 7), which could be used to distinguish different varieties. For instance, SSR231, SSR121 and SSR122, SSR121 and SSR125, SSR122 and SSR165, SSR122 and SSR191 can distinguish four different alfalfa varieties with 100% accuracy. SSR127 and SSR165 can distinguish LS from the other alfalfa varieties (Fig 7). Interestingly, these SSRs originated from transcript sequence of *LRR-RLK*, *PP2C12*, *PP2C25*, *ERF026*, *HSP70*, and *ARR5* genes (Table 4). *LRR-RLKs*, *PP2Cs*, *ERFs* and *HSPs* are important regulators of plant responses to abiotic stress. Transcriptome sequencing results showed that *PP2C12*, *PP2C25*, *ERF026* in roots, and *ARR5* in leaves were upregulated by NaCl treatment in GIB, while in LS, they were unchanged. Meanwhile, *LRR-RLK* was downregulated by NaCl treatment in LS, while in GIB, it was unchanged, *and HSP70* was upregulated by NaCl treatment in both varieties (S3 Table). Overexpression of *OsSTLK* (a member of LRR-RLK) (**Lin et al. 2021**), *PnLRR-RLK27* (**Wang et al. 2021**), *PagERF021* (**Fan et al. 2021**) and *MdHSP70–38* (**Han et al. 2021**) enhanced the salinity stress tolerance of transgenic plants through regulating ROS scavenging system. Overexpression of *AtARR22* (**Kang et al. 2021**) and *AtARR5* (**Shi et al. 2021**) genes in *Arabidopsis* enhanced the freezing tolerance of transgenic lines by improving cell membrane integrity. *PP2CA* together with *ABI1* inhibits SnRK2.4 activity and regulates plant responses to salinity(**Krzywinska et al. 2021**), and overexpression of *BpPP2C1* in *Betula platyphylla* improved the salinity tolerance in transgenic lines, while knockout of *BpPP2C1* exhibited sensitive to salt stress (**Xing et al. 2021**). Therefore, we speculate that these genes may serve as marker genes for distinguishing the four alfalfa varieties with contrasting salinity tolerance.

Meanwhile, the UPGMA clustering based on genetic distances, the six newly developed SSR markers to class four alfalfa varieties into three groups: group I (LS), group II (GIB and GN5), and group III (GN3) (Fig 8). This finding is similar to our previous results on salinity tolerance in alfalfa, which categorized the varieties into three groups: high salinity tolerance (GIB and GN5), salinity sensitivity (GN3), and high salinity sensitivity (LS) (**Yu et al. 2021**; **Yu et al. 2022**). The combination of salt tolerance, PCR, and cluster analyses indicated a potential association between the six newly developed SSR markers and salinity tolerance in alfalfa.

**References**

Yu R, Wang G, Yu X, Li L, Li C, Song Y, Xu Z, Zhang J, Guan C. **Assessing alfalfa (*Medicago sativa* L.) tolerance to salinity at seedling stage and screening of the salinity tolerance traits**. Plant Biol. 2021; 23(4): 664–674.

Yu R, Wang X, Wang G, Xu Z, Gao Q, Du X, zhang Y. **Analysis of salinity-tolerance and screening of salinity-tolerance evaluation indicators in *Medicago sativa* L. varieties at seedling stage**. Acta Agrestia Sinica 2022; 30(7): 1781–1789.

Lin F, Li S, Wang K, Tian H, Gao J, Zhao Q, Du C. **A leucine-rich repeat receptor-like kinase, OsSTLK, modulates salt tolerance in rice**. Plant Sci. 2020; 296: 110465. 42.

Wang J, Liu S, Li C, Wang T, Zhang P, Chen K. **PnLRR-RLK27, a novel leucine-rich repeats receptor-like protein kinase from the Antarctic moss *Pohlia nutans,* positively regulates salinity and oxidation-stress tolerance**. Plos One. 2017; 12(2): e0172869.

Fan G, Gao Y, Wu X, Yu Y, Yao W, Jiang J, Liu H, Jiang T. **Functional analysis of PagERF021 gene in salt stress tolerance in *Populus alba* × *P. glandulosa***. Plant Genome. 2024; 17(4): e20521.

Han X, Song C, Fang S, Wei Y, Tian J, Zheng X, Jiao J, Wang M, Zhang K, Hao P, et al. **Systematic identification and analysis of the HSP70 genes reveals MdHSP70-38 enhanced salt tolerance in transgenic tobacco and apple**. Int J Biol Macromol. 2025; 289: 138943.

Kang N, Cho C, Kim J. **Inducible expression of Arabidopsis response regulator 22 (ARR22), a Type-C ARR, in transgenic *Arabidopsis* enhances drought and freezing tolerance**. PLoS One. 2013; 8(11): e79248.

Shi Y, Tian S, Hou L, Huang X, Zhang X, Guo H, Yang S. **Ethylene signaling negatively regulates freezing tolerance by repressing expression of CBF and type-A ARR genes in *Arabidopsis***. Plant Cell. 2012; 24: 2578-2595.

Krzywińska E, Kulik A, Bucholc M, Fernandez MA, Rodriguez PL, Dobrowolska GY. **Protein phosphatase type 2C PP2CA together with ABI1 inhibits SnRK2.4 activity and regulates plant responses to salinity**. Plant Signal Behav*.* 2016; 11(12): e1253647.

Xing B, Gu C, Zhang T, Zhang Q, Yu Q, Jiang J, Liu G. **Functional study of *BpPP2C1* revealed its role in salt stress in *Betula platyphylla***. Front Plant Sci. 2021; 11: 617635.

In order to make the description more accurate and logical, in **lines 341-344**, the“For instance, SSR231, SSR121 and SSR122, SSR121 and SSR125, SSR122 and SSR165, SSR122 and SSR191 can distinguish four different alfalfa varieties with 100% accuracy. SSR127 and SSR165 can distinguish LS from the other alfalfa varieties (Fig 7)”**was moved to** the **Discussion** section in the revised manuscript (**Now in lines 456-459**).

The “Transcriptome sequencing results showed that *PP2C12*, *PP2C25*, *ERF026* in roots, and *ARR5* in leaves were upregulated by NaCl treatment in GIB, while in LS, they were unchanged. Meanwhile, *LRR-RLK* was downregulated by NaCl treatment in LS, while in GIB, it was unchanged, *and HSP70* was upregulated by NaCl treatment in both varieties (S3 Table). Overexpression of *OsSTLK* (a member of LRR-RLK) [41], *PnLRR-RLK27* [42], *PagERF021* [43], and *MdHSP70–38* [44] enhanced the salinity stress tolerance of transgenic plants through regulating ROS scavenging system. Overexpression of *AtARR22* [45] and *AtARR5* [46] genes in *Arabidopsis* enhanced the freezing tolerance of transgenic lines by improving cell membrane integrity. *PP2CA* together with *ABI1* inhibited SnRK2.4 activity and regulated plant responses to salinity[47], and overexpression of *BpPP2C1* in *Betula platyphylla* improved the salinity tolerance of transgenic lines, and knockout of *BpPP2C1* exhibited sensitive to salt stress [48]” **was added in** the **Discussion** section in the revised manuscript (**Now in lines 462-474**).

**Lines 458-462,** “This finding is similar to our previous study on salinity tolerance analysis of alfalfa: the salinity tolerance (GIB and GN5), salinity sensitivity (GN3), and high salinity sensitivity (LS) groups [5, 22], suggesting that there is a potential association between these six SSR markers and salinity tolerance in alfalfa.” **was revised as**

**“**This finding is similar to our previous results on salinity tolerance in alfalfa, which categorized the varieties into three groups: high salinity tolerance (GIB and GN5), salinity sensitivity (GN3), and high salinity sensitivity (LS) [5,22]. The combination of salt tolerance, PCR, and cluster analyses indicated a potential association between the six newly developed SSR markers and salinity tolerance in alfalfa.**”** in the revised manuscript (**Now in lines 486-491**).

**Reviewer 2:**

***The paper combines with transcriptome sequencing, provides a thorough and interesting exploration of key SSR loci and distribution characteristics of salt tolerance genes in alfalfa. The research question is well-defined and the methodology is sound. This finding not only provide an efficient tool for the large-scale development of markers related to specific traits, but also lays a foundation for genetic analysis in alfalfa. While the paper could benefit from further clarification or elaboration.***

**Response:** Many thanks for the reviewer’s comments.

***1. Some sentences in the current abstract are too long. It is suggested to simplify the sentences to make the information more direct and clear. At the same time,*** ***the results are introduced too much, and the main conclusions of the study should be highlighted.***

**Response:** Many thanks for the reviewer’s constructive suggestions. To polish the grammar and language, we have invited some colleagues who are skilled in English to check the language throughout the entire manuscript. These changes will not influence the content and framework of the MS. And here all changes made to the text are indicated in red color in the revised manuscript. Hopefully the language is now acceptable for publication.

Additionally, the results are introduced too much, some sentences such as **lines 244-249** “Of which, 430 (291 upregulated, 139 downregulated), 655 (464 upregulated, 191 downregulated), 407 (96 upregulated，311 downregulated), 152 (27 upregulated, 125 downregulated), 219 (130 upregulated, 89 downregulated), 409 (157 upregulated, 252 downregulated), 311 (99 upregulated，212 downregulated), 172 (99 upregulated, 73 downregulated) DEUs in the eight comparison groups, separately (Fig 3A and 3B).” **was revised as**

“Of which, 430, 655, 407, 152, 219, 409, 311, and 172 DEUs were identified in G_200_L_/G_0_L_, L_200_L_/L_0_L_, G_0_L_/L_0_L_, G_200_L_/L_200_L_, G_200_R_/G_0_R_, L_200_R_/L_0_R_, G_0_R_/L_0_R_, and G_200_R_/L_200_R_, respectively (Figs 3A and 3B)” (**Now in lines 258-260**)

**Lines 342-345**, “For instance, SSR231, SSR121 and SSR122, SSR121 and SSR125, SSR122 and SSR165, SSR122 and SSR191 can distinguish four different alfalfa varieties with 100% accuracy. SSR127 and SSR165 can distinguish LS from the other alfalfa varieties (Fig 7).” have been removed in the **Results** section, and **moved to** the **Discussion** section in the revised manuscript (**Now in lines 456-459**).

***2. The language expression of the article needs to be further improved. It is suggested to simplify the sentence structure, avoid repetition, select more accurate vocabulary and strengthen the transition and cohesion, so that the language expression of the article can become more fluent and easy to understand.***

**Response:** Many thanks for the reviewer’s constructive suggestions. To polish the grammar and language throughout the manuscript, we have invited some colleagues who are skilled in English to check the language. We have revised the whole manuscript carefully and tried to avoid any grammar or syntax error. All changes made to the text are indicated in red color in the revised manuscript. We hope that the language is now acceptable for publication.

***3. In the 127th line of the manuscript, the materials and methods are mentioned “Four alfalfa varieties differing in salinity tolerance ( GN5, GN3, LS and GIB) were selected based on previous studies”, 141 lines “. The 140 tissue of roots and leaves of GIB and LS were used for transcriptome sequencing …”, it is recommended to clarify the reasons for selecting only these two groups of samples and the specific salt tolerance of the four materials.***

**Response:** Thank you very much for reviewer’s constructive suggestions. Our previous studies showed that Gibraltar (GIB) and Gannong No.5 (GN5) were high salinity-tolerant varieties, Gannong No.3 (GN3) was a salinity-sensitivity variety, and LS1405 (LS) was a variety of high salinity sensitivity (**Yu et al. 2021**; **Yu et al. 2022**). Therefore, two varieties with significant differences in salt tolerance, namely, GIB and LS, were used for transcriptome sequencing to identify key genes related to salinity stress tolerance and develop SSR markers linked to this trait.

Transcriptome sequencing has been effectively and rapidly utilized for the development of SSR marker in plants. SSRs can realize targeted markers directly related to target traits. Therefore, we developed SSR markers related to salinity tolerance based on the transcriptome sequencing data. Moreover, to verify whether the developed SSR markers can be applied to the identification of salt tolerance traits of alfalfa, two salinity-tolerant varieties (GIB, GN5) and two salinity-sensitive varieties (GN3, LS) were selected to validate the newly developed SSR markers.

**References**

Yu R, Wang G, Yu X, Li L, Li C, Song Y, Xu Z, Zhang J, Guan C. **Assessing alfalfa (*Medicago sativa* L.) tolerance to salinity at seedling stage and screening of the salinity tolerance traits**. Plant Biol. 2021; 23(4): 664–674.

Yu R, Wang X, Wang G, Xu Z, Gao Q, Du X, zhang Y. **Analysis of salinity-tolerance and screening of salinity-tolerance evaluation indicators in *Medicago sativa* L. varieties at seedling stage**. Acta Agrestia Sinica 2022; 30(7): 1781–1789.

Furthermore, according to your suggestions, we have added the reasons for variety selection in the manuscript. The corresponding sentences of the ***Methods*** (**Now in** **lines 128-135, 150-153**) section have been rewritten in the revised manuscript.

***4. It is recommended to clarify the reasons for supplementing the use of 0 and 200 mM*** ***NaCl treatments for alfalfa seedlings.***

**Response:** Thank you very much for reviewer’s constructive suggestions. According to your suggestions, “The NaCl concentrations were set according to the results of a preliminary experiment, which showed a significant difference in growth between GIB and LS under 200 mM NaCl treatment” **have** **been added in** **Methods** in the revised manuscript (**Now in lines 147-150**).

**Reviewer 3:**

***The study presents a valuable approach to developing trait-associated SSR markers in alfalfa using transcriptome data. However, significant revisions are required to meet the standards of PLOS ONE.***

**Response:** Many thanks for the reviewer’s comments.

**Key Points for Revision:
*1. Number of Biological Replicates: The use of only two biological replicates for transcriptome sequencing is a major methodological flaw. Three or more replicates are standard for robust statistical analysis in RNA-seq studies. This limitation must be addressed and justified in the manuscript.***

**Response:** We would like to thank the reviewer for pointing out this issue. In the present study, to reveal the distribution characteristics of SSR loci, and to explore the key SSR loci related to salinity-tolerant gene (mRNA) in alfalfa, we analyzed the roots and leaves transcriptome of GIB (salinity-tolerant) and LS (salinity-sensitive) under 0 and 200 mM NaCl treatments from two biological replicate RNA samples. Just as the reviewer’s comments, recently, researchers are increasingly realizing the importance of biological replicates for assessing statistical confidence in RNA-seq experiments. However, to our knowledge, some mRNAs related research papers, two libraries were prepared under one certain condition to identify mRNAs relative to growth and development, and response to various stresses in plant. Then, some further analysis works such as qRT-PCR would be performed to validate the mRNA expression profiles from the sequencing analysis (**Du et al. 2021, Cao et al. 2019; Chen et al. 2019; Xu et al. 2015**). Moreover, some studies conducted two or three biological replicates for each cDNA library to do digital gene expression (DGE) tag profiling studies (**Li et al. 2014; Saminathan et al. 2014**). Nevertheless, the results showed that the number of raw and clean tags generated from different replicates for one library was very similar, resulting in no significant differences observed between the number and annotation of assembled unigenes and DGEs (**Li et al. 2014**). Overall, these results indicated that the quality and quantity of RNA-seq map reads from two libraries could meet the acceptable range for further analysis. We addressed other reasons as follows:

As previously discussed, next-generation sequencing has been adapted to the study of a wide range of nucleic acid populations, such as mRNA (RNA-seq), small RNA (sRNA) (**McCormick et al. 2011**). These applications vary with regard to the templates used, but they rely on the same sequencing technologies. Several reports have used technical replicates, that is, the same library was sequenced for multiple times to demonstrate the high reliability of Illumina sequencing, the results indicated that the Illumina sequencing data were highly replicable, and thus, for many purposes, it may suffice to sequence each mRNA sample only twice (**Marioni et al. 2008; Linsen et al. 2009; Fahlgren et al. 2009**).

Furthermore, the SSRs mainly includes two categories, namely genomic SSRs and expressed sequence tags SSR, which are originated from genomic sequences and transcriptome sequences, respectively (**Shi et al. 2022; Wu et al. 2014**). Compared with the traditional mining SSRs from genomic sequences, the developed SSR markers from transcriptome sequences was considered as a simple and effective method with lower cost and less labor (**Pan et al. 2024; Squirrell et al. 2003**). Currently, the discovery and mining of SSR loci through transcriptome sequences have been successfully applied to many plant species (**Jiao et al. 2024; Liu et al. 2022**). However, the development of trait-associated SSR markers in alfalfa through transcriptome sequence is rarely reports.

In this study, to guarantee the accuracy and consistency for SSR loci information for each sample and avoid the type of possible error in sampling, 16 libraries were constructed from a proportion of the composite root/leaf sample under 0 and 200 mM NaCl treatments, respectively. For composite sample preparation, equal amount of root/leaf samples from four randomly selected individual plants (per pot) from each treatment respectively were pooled and collected as a replicate. We try to reduce the possible error in sampling. Finally, one cDNA library as a representative of the corresponding population at each treatment was constructed and sequenced for the further analysis.

**References**

Du, X., Yu, R., Shi C., et al. **Comparative transcriptomic reveals osmotic and ionic stress key genes contributing to the difference in the salinity tolerance of two pak choi cultivars**. Environ. Exp. Bot. 2021, 191, 104621

Cao, Q., Xv, C., Jiang, Q. et al. **Comparative transcriptome analysis reveals key genes responsible for the homeostasis of iron and other divalent metals in peanut roots under iron deficiency**. Plant Soil, 2019, 445, 513–531.

Chen, C., Cao, Q., Jiang, Q., et al. **Comparative transcriptome analysis reveals gene network regulating cadmium uptake and translocation in peanut roots under iron deficiency**. BMC Plant Biol. 2019, 19.DOI:10.1186/s12870-019-1654-9.

Xu, L., Wang, Y., Liu, W, et al. **De novo sequencing of root transcriptome reveals complex cadmium-responsive regulatory networks in radish (*Raphanus sativus* L.)**.Plant Sci. 2015, 236, 313-323.

Li, J., Liu, B., Cheng, F., et al. **Expression profiling reveals functionally redundant multiple-copy genes related to zinc, iron and cadmium responses in *Brassica rapa***. New Phytol. **2014**, 182-194.

Saminathan, T., Nimmakayala, P., Manohar, S., et al. **Differential gene expression and alternative splicing between diploid and tetraploid watermelon**. J. Exp. Bot. 2014, **66**, 1369-1385.

McCormick, K., Willmann, M., Meyers, B. **Experimental design, preprocessing, normalization and differential expression analysis of small RNA sequencing experiments**. Silence, 2011, 2. https://doi.org/10.1186/1758-907X-2-2.

Marioni, J., Mason, C., Mane, S., et al. **RNA-seq: an assessment of technical reproducibility and comparison with gene expression arrays**. Genome Res. 2008, 18, 1509-1517.

Linsen, S., de Wit, E., Janssens, G., et al. **Limitations and possibilities of small RNA digital gene expression profiling**. Nat. Methods 2009, 6, 474-476

Fahlgren, N., Sullivan, C., Kasschau, K., et al. **Computational and analytical framework for small RNA profiling by high-throughput sequencing**. RNA 2009, 15, 992-1002.

Shi, Z., Zhao, W., Li, Z., et al. **Development and validation of SSR markers related to flower color based on full-length transcriptome sequencing in *Chrysanthemum***. Sci. Rep. 2022, *12*, 22310.

Wu, H.B., Gong, H., Liu, P., et al. **Large-scale development of EST-SSR markers in sponge gourd via transcriptome sequencing**. Mol. Breeding 2014, *34*, 1903–1915.

Pan, G., Xie, J., Qin, Y., et al. **Development of SSR markers for genetic diversity analysis and species identification in *Polygonatum odoratum* (Mill.) Druce based on transcriptome sequences**. PLoS One 2024, *19*, e0308316.

Squirrell, J., Hollingsworth, P., Woodhead, M., et al. **How much effort is required to isolate nuclear microsatellites from plants?** Mol. Ecol. 2003, *12*, 1339–1348.

Jiao, L., Han, C., Zhu, J., et al. **Transcriptome analysis and development of EST-SSR markers in the mushroom *Auricularia heimuer***. Sci. Rep. 2024, *14*, 12340.

Liu, Y., Fang, X., Tang, T., et al. **Inflorescence transcriptome sequencing and development of new EST-SSR markers in common buckwheat (*Fagopyrum esculentum*)**. Plants 2022, *11*, 742.

In addition, according to the reviewer’s suggestion, to make the description of samples collection of transcriptome sequencing clearer and accurately, the corresponding revisions had been made as follows.

**Lines 139-143**, “Following 14 days of treatment, roots and leaves samples were flash-frozen in liquid nitrogen and stored at -80°C...” **was revised as**

“Four weeks after sowing, root and leaf samples of GIB and LS were selected for transcriptome sequencing. Moreover, the leaf samples of GIB, GN5, GN3 and LS under NaCl-free treatment were selected to validate the newly developed SSR markers. All collected samples were flash-frozen in liquid nitrogen and stored at −80°C. Equal amount of root/leaf samples from four randomly selected individual plants (per pot) from each treatment were pooled and collected as a replicate. Two biological replicates were analyzed for transcriptome sequencing.” in the revised manuscript (**Now in lines 150-156**).

***2. Inconsistency in Data: The description of the cultivar clustering in the abstract is incorrect and contradicts the results presented in the main text and Figure 8. This must be corrected for consistency.***

**Response:** We were really sorry for our careless mistakes. Thank you for kindly reminding us. According to the reviewer’s suggestions, in **Abstract**, “the salinity sensitive variety GN3 in the group I, salinity tolerance varieties GIB and GN5 were in the group II, and high sensitive variety LS in the group III” **was revised as**

“The high salinity-sensitive variety LS was placed in group I, the high tolerant varieties GIB and GN5 formed group II, and the sensitive variety GN3 was included in group III” in **Abstract** section in the revised manuscript (**Now in lines 66-68**)

Furthermore, the Figure 8 was also corrected in the revised-Figure 8.

***3. Low Primer Validation Success:*** ***Out of 211 primer pairs designed from salinity-responsive genes, only seven were found to be clearly polymorphic. The authors should discuss the potential reasons for this very low success rate (e.g., tetraploid genome complexity, assembly errors) in the Discussion section.***

**Response:** Many thanks for the reviewer’s comments. In this study, among the 211 pairs of primers, not only 7 pairs amplified polymorphism, but also 110 pairs of primers could detect the amplified products, including polymorphic markers. However, to make the data more accurate and reliable, 7 out of 110 pairs of primers (SSR121, SSR122, SSR125, SSR127, SSR165, SSR191 and SSR231) had clear amplified bands were selected, and the amplified bands each of those are different in GN3, GN5, GIB and LS. The development of a small number of SSR based on transcriptome data can realize targeted markers directly related to target traits. For example, Xia et al. (2014) developed 191 SSR markers based on the transcriptome sequences and found nine SSR markers significantly associated with plant height through association analysis in coconut. Shi et al. (2022) identified 11,699 SSR loci and developed eight polymorphic SSR markers related to flower color.

**References**

Xia W, Xiao Y, Liu Z, et al. **Development of gene-based simple sequence repeat markers for association analysis in *Cocos nucifera***. *Mol. Breeding*, 2014, 34, 525–535.

Shi Z, Zhao W, Li Z, et al. **Development and validation of SSR markers related to flower color based on full-length transcriptome sequencing in *Chrysanthemum***. Sci Rep*.* 2022; 12(1): 22310.

In addition, to make the description more clearly and accurately, the corresponding sentences of the ***Results*** (**Now in** **lines 351-355**) and **Discussion** (**Now in** **lines 453-459**) sections have been rewritten in the revised manuscript.

***4. Limited Validation Scope: The new markers were validated using only four alfalfa varieties. This is a very small sample size to claim broad utility. This should be acknowledged as a significant limitation of the study.***

**Response:** We thank the reviewer for bringing this to our attention. The tested varieties are salt-tolerant and salt-sensitive varieties identified from the analysis of salt-tolerant characteristics of more than 30 varieties (**Yu et al. 2021**; **Yu et al. 2022**), which are representative to some extent. Our main purpose here is to validate whether the newly developed salt-tolerant markers can distinguish the various salinity tolerance alfalfa varieties and whether they are related to salt tolerance. Moreover, just as the reviewer’s comments, if the number of samples is too small, it may also lead to inaccurate results of cluster analysis. However, cross-validation can make full use of the data and improve the accuracy of analysis with a small sample size. In this study, PCR analysis was carried out to detect the amplification products of 7 newly developed SSR markers (SSR121, SSR122, SSR125, SSR127, SSR165, SSR191 and SSR231). The amplified bands each of those are different in GN3, GN5, GIB and LS, which could be used to distinguish different varieties. For instance, SSR231, SSR121 and SSR122, SSR121 and SSR125, SSR122 and SSR165, SSR122 and SSR191 can distinguish four different alfalfa varieties with 100% accuracy. SSR127 and SSR165 can distinguish LS from the other alfalfa varieties. Meanwhile, through UPGMA clustering based on genetic distances, the six newly developed SSR markers classified four alfalfa varieties into three groups: group I (LS), group II (GIB and GN5), and group III (GN3) (Fig 8). This finding is similar to our previous results on salinity tolerance in alfalfa, which categorized the varieties into three groups: high salinity tolerance (GIB and GN5), salinity sensitivity (GN3), and high salinity sensitivity (LS) (**Yu et al. 2021**; **Yu et al. 2022**). The combination of salt tolerance, PCR, and cluster analyses indicated a potential association between the six newly developed SSR markers and salinity tolerance in alfalfa.

**References**

Yu R, Wang G, Yu X, Li L, Li C, Song Y, Xu Z, Zhang J, Guan C. **Assessing alfalfa (*Medicago sativa* L.) tolerance to salinity at seedling stage and screening of the salinity tolerance traits**. Plant Biol. 2021; 23(4): 664–674.

Yu R, Wang X, Wang G, Xu Z, Gao Q, Du X, zhang Y. **Analysis of salinity-tolerance and screening of salinity-tolerance evaluation indicators in *Medicago sativa* L. varieties at seedling stage**. Acta Agrestia Sinica 2022; 30(7): 1781–1789.

In order to make the description more accurate and logical, in **lines 341-344**, the“For instance, SSR231, SSR121 and SSR122, SSR121 and SSR125, SSR122 and SSR165, SSR122 and SSR191 can distinguish four different alfalfa varieties with 100% accuracy. SSR127 and SSR165 can distinguish LS from the other alfalfa varieties (Fig 7)”**was moved to** the **Discussion** section in the revised manuscript (**Now in lines 456-459**).

The “Transcriptome sequencing results showed that *PP2C12*, *PP2C25*, *ERF026* in roots, and *ARR5* in leaves were upregulated by NaCl treatment in GIB, while in LS, they were unchanged. Meanwhile, *LRR-RLK* was downregulated by NaCl treatment in LS, while in GIB, it was unchanged, *and HSP70* was upregulated by NaCl treatment in both varieties (S3 Table). Overexpression of *OsSTLK* (a member of LRR-RLK) [41], *PnLRR-RLK27* [42], *PagERF021* [43], and *MdHSP70–38* [44] enhanced the salinity stress tolerance of transgenic plants through regulating ROS scavenging system. Overexpression of *AtARR22* [45] and *AtARR5* [46] genes in *Arabidopsis* enhanced the freezing tolerance of transgenic lines by improving cell membrane integrity. *PP2CA* together with *ABI1* inhibited SnRK2.4 activity and regulated plant responses to salinity[47], and overexpression of *BpPP2C1* in *Betula platyphylla* improved the salinity tolerance of transgenic lines, and knockout of *BpPP2C1* exhibited sensitive to salt stress [48]” **was added in** the **Discussion** section in the revised manuscript (**Now in lines 462-474**).

**Lines 458-462,** “This finding is similar to our previous study on salinity tolerance analysis of alfalfa: the salinity tolerance (GIB and GN5), salinity sensitivity (GN3), and high salinity sensitivity (LS) groups [5, 22], suggesting that there is a potential association between these six SSR markers and salinity tolerance in alfalfa.” **was revised as**

**“**This finding is similar to our previous results on salinity tolerance in alfalfa, which categorized the varieties into three groups: high salinity tolerance (GIB and GN5), salinity sensitivity (GN3), and high salinity sensitivity (LS) [5,22]. The combination of salt tolerance, PCR, and cluster analyses indicated a potential association between the six newly developed SSR markers and salinity tolerance in alfalfa.**”** in the revised manuscript (**Now in lines 486-491**).

***5. Discussion of Limitations: The Discussion section needs to be strengthened by transparently addressing the limitations mentioned above (number of replicates, primer success rate, and small validation set).***

**Response:** We thank the reviewer for bringing this to our attention. In this study, not only 7 of 211 pairs of primers amplified polymorphism, but 110 pairs of primers amplified the corresponding products. However, to make the data more accurate and reliable, 7 out of 110 pairs of primers (SSR121, SSR122, SSR125, SSR127, SSR165, SSR191 and SSR231) had clear amplified band were selected, and the amplified bands each of those are different in GN3, GN5, GIB and LS. The development of a small number of SSR based on transcriptome data can realize targeted markers directly related to target traits. For example, Xia et al. (2014) developed 191 SSR markers based on the transcriptome sequences and found nine SSR markers significantly associated with plant height through association analysis in coconut. Shi et al. (2022) identified 11,699 SSR loci and developed eight polymorphic SSR markers related to flower color.

In addition, the tested varieties are salt-tolerant and salt-sensitive varieties identified from the analysis of salt-tolerant characteristics of more than 30 varieties (**Yu et al. 2021**; **Yu et al. 2022**), which are representative to some extent. Our main purpose here is to validate whether the newly developed salt-tolerant markers can distinguish the various salinity tolerance alfalfa varieties and whether they are related to salt tolerance. Moreover, just as the reviewer’s comments, if the number of samples is too small, it may also lead to inaccurate results of cluster analysis. However, cross-validation can make full use of the data and improve the accuracy of analysis with a small sample size. In this study, PCR analysis was carried out to detect the amplification products of 7 newly developed SSR markers (SSR121, SSR122, SSR125, SSR127, SSR165, SSR191 and SSR231). The amplified bands each of those are different in GN3, GN5, GIB and LS (Fig 7), which could be used to distinguish different varieties. For instance, SSR231, SSR121 and SSR122, SSR121 and SSR125, SSR122 and SSR165, SSR122 and SSR191 can distinguish four different alfalfa varieties with 100% accuracy. SSR127 and SSR165 can distinguish LS from the other alfalfa varieties (Fig 7). Meanwhile, the UPGMA clustering based on genetic distances, the six newly developed SSR markers to class four alfalfa varieties into three groups: group I (LS), group II (GIB and GN5), and group III (GN3) (Fig 8). This finding is similar to our previous study on salinity tolerance analysis of alfalfa: the high salinity tolerance (GIB and GN5), salinity sensitivity (GN3), and high salinity sensitivity (LS) groups (**Yu et al. 2021**; **Yu et al. 2022**). Combination of salt tolerance, PCR, and cluster analyses indicated that there is a potential association between these six SSR markers and salinity tolerance in alfalfa.

**References**

Xia W, Xiao Y, Liu Z, et al. **Development of gene-based simple sequence repeat markers for association analysis in *Cocos nucifera***. *Mol. Breeding*, 2014, 34, 525–535.

Shi Z, Zhao W, Li Z, et al. **Development and validation of SSR markers related to flower color based on full-length transcriptome sequencing in *Chrysanthemum***. Sci Rep*.* 2022; 12(1): 22310.

Yu R, Wang G, Yu X, Li L, Li C, Song Y, Xu Z, Zhang J, Guan C. **Assessing alfalfa (*Medicago sativa* L.) tolerance to salinity at seedling stage and screening of the salinity tolerance traits**. Plant Biol. 2021; 23(4): 664–674.

Yu R, Wang X, Wang G, Xu Z, Gao Q, Du X, zhang Y. **Analysis of salinity-tolerance and screening of salinity-tolerance evaluation indicators in *Medicago sativa* L. varieties at seedling stage**. Acta Agrestia Sinica 2022; 30(7): 1781–1789.

***6. Elaboration on Functional Links: The paper successfully links the markers to important stress-response genes like PP2C, ERF026, and HSP70. The Discussion should be expanded to elaborate more on the known roles of these specific genes in salinity tolerance, thereby reinforcing the potential value of the developed markers.***

**Response:** Thanks for the reviewer’s helpful suggestion. According to the reviewer’s suggestions, in **Discussion**, “Moreover, the *LRR-RLKs* [41], *PP2Cs* [42], *ERFs* [43], *HSP70* [44], and *ARRs* [45] genes have been found to be involved in the abiotic stress response and tolerance of plants, especially during salinity stress.” **was revised as**

**“***LRR-RLKs*, *PP2Cs*, *ERFs*, and *HSPs* are important regulators of plant responses to abiotic stress. Transcriptome sequencing results showed that *PP2C12*, *PP2C25*, *ERF026* in roots, and *ARR5* in leaves were upregulated by NaCl treatment in GIB, while in LS, they were unchanged. Meanwhile, *LRR-RLK* was downregulated by NaCl treatment in LS, while in GIB, it was unchanged, *and HSP70* was upregulated by NaCl treatment in both varieties (S3 Table). Overexpression of *OsSTLK* (a member of LRR-RLK) [41], *PnLRR-RLK27* [42], *PagERF021* [43], and *MdHSP70–38* [44] enhanced the salinity stress tolerance of transgenic plants through regulating ROS scavenging system. Overexpression of *AtARR22* [45] and *AtARR5* [46] genes in *Arabidopsis* enhanced the freezing tolerance of transgenic lines by improving cell membrane integrity. *PP2CA* together with *ABI1* inhibited SnRK2.4 activity and regulated plant responses to salinity[47], and overexpression of *BpPP2C1* in *Betula platyphylla* improved the salinity tolerance of transgenic lines, and knockout of *BpPP2C1* exhibited sensitive to salt stress [48]. Therefore, we speculate that these genes may serve as marker genes for distinguishing the four alfalfa varieties with contrasting salinity tolerance.**”** in the revised manuscript (**Now in Lines 461-476**).

**Furthermore,** three literatures “[41], [46] and [48]” related to this study **were added** in the references part. Additionally, the orders of other literatures in this MS were changed accordingly (**please see the revised references**).
