# Supplementary material for: Development of SSR markers related to salinity resistance based on transcriptomic sequences in Medicago sativa
Source: PLoS One. 2025 Nov 14;20(11):e0336528. doi: 10.1371/journal.pone.0336528 (PMC12617910; doi:10.1371/journal.pone.0336528)
Supplement: Attachment — Submitted filename: Referee report.docx [file pone.0336528.s015.docx]

Recommendation: Major Revision

The study presents a valuable approach to developing trait-associated SSR markers in alfalfa using transcriptome data. However, significant revisions are required to meet the standards of PLOS ONE.

Key Points for Revision:

- Number of Biological Replicates: The use of only two biological replicates for transcriptome sequencing is a major methodological flaw. Three or more replicates are standard for robust statistical analysis in RNA-seq studies. This limitation must be addressed and justified in the manuscript.
- Inconsistency in Data: The description of the cultivar clustering in the abstract is incorrect and contradicts the results presented in the main text and Figure 8. This must be corrected for consistency.
- Low Primer Validation Success: Out of 211 primer pairs designed from salinity-responsive genes, only seven were found to be clearly polymorphic. The authors should discuss the potential reasons for this very low success rate (e.g., tetraploid genome complexity, assembly errors) in the Discussion section.
- Limited Validation Scope: The new markers were validated using only four alfalfa varieties. This is a very small sample size to claim broad utility. This should be acknowledged as a significant limitation of the study.
- Discussion of Limitations: The Discussion section needs to be strengthened by transparently addressing the limitations mentioned above (number of replicates, primer success rate, and small validation set).
- Elaboration on Functional Links: The paper successfully links the markers to important stress-response genes like PP2C, ERF026, and HSP70. The Discussion should be expanded to elaborate more on the known roles of these specific genes in salinity tolerance, thereby reinforcing the potential value of the developed markers.
